# SEMA6A inhibits tumor progression and boosts anti-tumor immunity *via* blocking the ISG15/TGFβ axis in colorectal cancer

**DOI:** 10.1186/s43556-026-00493-6

**Published:** 2026-06-17

**Authors:** Fang Zhang, Rixin Zhang, Zheng Yan, Jinbao Zong, Mingxuan Zhou, Tiegang Li, Yufang Hou, Silin Lv, Zifan Zeng, Wenyi Zhao, Yixin Zhou, Zengni Zhu, Siying Huang, Min Yang

**Affiliations:** 1 State Key Laboratory of Digestive Health, Institute of Materia Medica, Chinese Academy of Medical Sciences and Peking Union Medical College, No. 2 Nanwei Road, Beijing, 100050 China; 2 State Key Laboratory of Bioactive Substance and Function of Natural Medicines, Institute of Materia Medica, Chinese Academy of Medical Sciences and Peking Union Medical College, Beijing, 100050 China; 3 Clinical Laboratory, The Affiliated Hospital of Qingdao University, Qingdao, 266000 China; 4 Qingdao Hospital of Traditional Chinese Medicine, The Affiliated Qingdao Hiser Hospital of Qingdao University, Qingdao, 266033 China

**Keywords:** Colorectal cancer, SEMA6A, Prognosis, Immunotherapy, ISG15

## Abstract

Semaphorins (SEMAs) comprise a diverse family of membrane-associated proteins whose dysregulation is implicated in various cancers, influencing tumor progression and immune responses. However, the role of SEMAs in colorectal cancer (CRC) remains underexplored. This study demonstrates that Semaphorin 6A (SEMA6A) is downregulated in CRC tissues, with low SEMA6A expression correlating with increased tumor aggressiveness and poor prognosis in CRC patients. Functional assays illustrate that SEMA6A overexpression exerts anti-tumor effects both in vitro and in vivo, while integrated RNA-seq and proteomic analyses identify ISG15 as a key downstream effector negatively regulated by SEMA6A. Mechanistically, SEMA6A inhibits the JAK-STAT3 pathway, leading to decreased ISG15 expression, which in turn abrogates the ISGylation-dependent stabilization of TGF-β1 and promotes its degradation, as confirmed by molecular docking, co-immunoprecipitation, and cycloheximide chase assays. Consequently, in vitro co-culture assays and in vivo experiments reveal that SEMA6A overexpression in tumor cells enhances CD8^+^ T cell cytotoxicity via blocking the ISG15/TGFβ axis. Bioinformatics analyses indicate that patients with CRC exhibiting high SEMA6A and low ISG15 expression show improved responses to immunotherapy. Importantly, SEMA6A overexpression reshapes the tumor microenvironment, boosts anti-tumor immunity of CD8^+^ T cells and inhibits immune evasion, thereby potentiating anti-PD-1 immunotherapy in vivo*.* Collectively, these findings highlight the potential of SEMA6A as a dual therapeutic strategy in CRC: inhibiting cancer progression while enhancing anti-tumor immunity by targeting the ISG15/TGF-β1 axis. Thus, SEMA6A represents both a prognostic indicator and a promising target to improve immunotherapy efficacy and enable personalized treatment strategies in CRC.

**Supplementary Information:**

The online version contains supplementary material available at 10.1186/s43556-026-00493-6.

## Introduction

Colorectal cancer (CRC) is a leading cause of cancer-related mortality worldwide [1, 2]. Early-stage CRC is often asymptomatic, resulting in frequent diagnoses at advanced stages. Early detection and surgical resection significantly improve prognosis, with a 5-year survival rate exceeding 90% for early stages, compared to much lower rates in advanced stages. Consequently, early detection, diagnosis, and intervention are pivotal in CRC management [3]. The advent of multi-omics technologies has enabled the identification of numerous biomarkers, making the discovery of highly specific and sensitive biomarkers essential for effective early screening and diagnosis of CRC.

The tumor microenvironment (TME) of CRC comprises a complex network of non-cellular elements, immune cells, mesenchymal stromal cells, and tumor cells, all of which interact dynamically to drive CRC progression [4]. Among these, the immune microenvironment plays a pivotal role and is characterized by significant heterogeneity, influenced by factors such as immune cell infiltration and neoantigen burden [5, 6]. High immune cell infiltration has been associated with improved prognosis and reduced recurrence rates in patients with CRC [7]. Consequently, TME-targeted immunotherapy has emerged as a promising treatment strategy [8]. However, clinical trials conducted between 2010 and 2013 showed limited response rates to immunotherapy in the general CRC population [9–13]. While patients with microsatellite instability-high (MSI-H) tumors exhibit enhanced responsiveness, the majority of patients with CRC show poor or no response to immunotherapy. Therefore, identifying biomarkers that can accurately predict immunotherapy response is essential for optimizing treatment decisions.

Semaphorins (SEMAs), initially recognized as axon guidance factors, have gained increasing attention for their role in tumorigenesis and progression. Specific SEMAs have been shown to correlate with prognosis and the immune microenvironment in CRC. For instance, elevated SEMA6B expression promotes an immunosuppressive TME in CRC [14]. SEMA6A, a member of the same family as SEMA6B, acts as a transmembrane protein signaling through the plexin A2 and plexin A4 receptors [15, 16]. SEMA6A exhibits a dual role in tumor regulation: its recombinant SEMA6A-1 domain inhibits growth factors and tumor neovascularization, suggesting anti-tumor potential [17]. Conversely, in BRAF-mutant melanoma, deletion of SEMA6A reduced tumor aggressiveness, while overexpression enhanced it [18]. Furthermore, a three-gene prognostic model for patients with MSI gastric cancer included SEMA6A, revealing a strong link between SEMA6A expression and immune cell infiltration. This study suggests that SEMA6A modulates MSI status and the immune microenvironment, thereby influencing survival and prognosis in gastric cancer [19]. Despite these observations, the precise role and underlying mechanisms of SEMA6A in CRC remain unclear.

The ongoing exploration of novel therapeutic strategies for CRC encompasses both the optimization of existing treatments and the discovery of new molecular targets. Recent advances include chemosensitizing agents such as Huachansu injection, which enhances the efficacy of irinotecan in CRC while reducing its intestinal toxicity [20]. Natural products like cycloastragenol also exert antitumor effects across multiple cancer types including CRC [21]. Despite these advances, the roles of endogenous regulators such as SEMAs in CRC remain largely unexplored. Here, we identify SEMA6A as a tumor suppressor in CRC. Elevated SEMA6A expression predicts favorable prognosis and serves as an independent prognostic factor. Mechanistically, SEMA6A inhibits the JAK-STAT3 pathway, leading to reduced ISG15 expression. ISG15 stabilizes TGF-β1 via ISGylation, and SEMA6A overexpression decreases TGF-β1 secretion, thereby promoting CD8⁺ T cell activation and cytotoxicity. In vivo, SEMA6A overexpression suppresses tumor growth, especially in immunocompetent mice, and synergizes with anti-PD-1 therapy. Collectively, these findings highlight the SEMA6A/ISG15/TGF-β1 axis as a potential therapeutic target for CRC immunotherapy.

## Results

### SEMA6A is downregulated in CRC, suppresses malignancy, and predicts poor prognosis

**Fig. 1:**
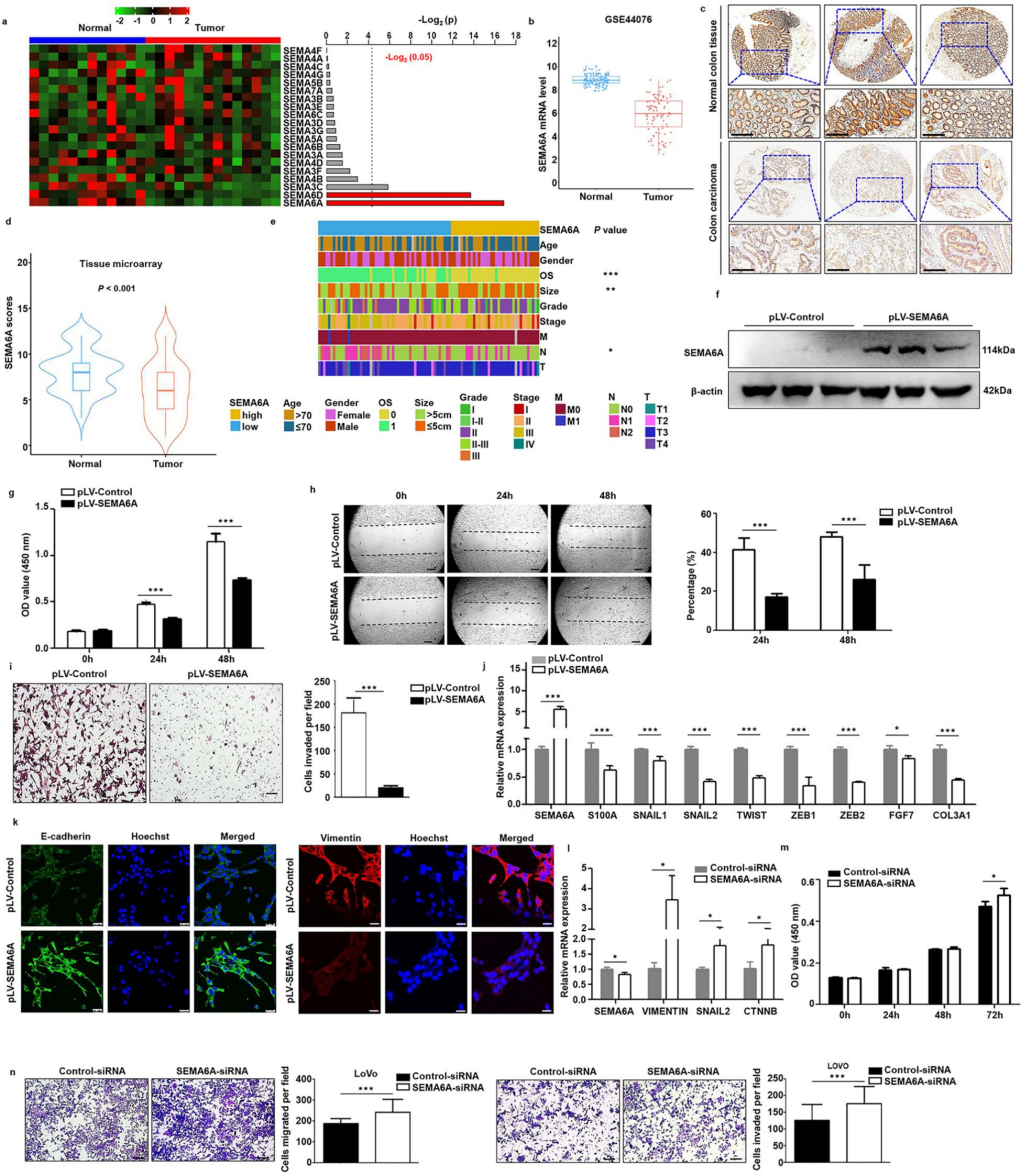
Expression of SEMA6A and In vitro validation of SEMA6A’s effect in CRC. **a** mRNA expression and differential expression of the semaphorin family in CRC and adjacent tissues as detected by NanoString technology. **b** SEMA6A expression in CRC tissues and adjacent normal tissues from the GSE44076 dataset. **c** Immunohistochemical staining of SEMA6A in colon cancer tissue microarray (scale bar, 200 μm). **d** Quantitative analysis of SEMA6A protein expression scores from the immunohistochemical staining of the TMA. **e** Heat map of correlation between SEMA6A and clinicopathological features in tissue microarray. **f** Western blot analysis of SEMA6A protein expression in the overexpressing SL4 cell line. **g** CCK-8 assay assessing the viability of control and pLV-SEMA6A CRC cells. **h** Wound healing assay to evaluate the migration ability of control and pLV-SEMA6A CRC cells (scale bar, 200 μm). **i** Transwell assay to assess the invasive capacity of control and pLV-SEMA6A CRC cells (scale bar, 200 μm). **j**-**k** RT-qPCR (**j**) and immunofluorescence (**k**) analysis of EMT-related biomarkers in control and pLV-SEMA6A CRC cells (scale bar, 25 μm). **l** RT-qPCR analysis of SEMA6A knockdown efficiency and EMT marker expression in negative control and SEMA6A-siRNA-transfected LoVo cells. **m**–**n** Assessment of proliferation (**m**), migration, and invasion abilities (**n**) of LoVo cells after SEMA6A knockdown (scale bar, 200 μm) (*P < 0.05, ***P < 0.001)

The NanoString assay was conducted to evaluate the expression levels of SEMAs in CRC, revealed that SEMA6A mRNA was the most significantly downregulated SEMA in CRC (Fig. 1a), confirmed by RNA-Seq data from the Gene Expression Omnibus (GEO) dataset GSE44076 (Fig. 1b). Immunohistochemistry (IHC) staining for SEMA6A was performed on a CRC tissue microarray (TMA) (Fig. 1c). The results indicated a substantial reduction in SEMA6A protein levels in carcinoma tissues compared to adjacent normal tissues (Fig. 1d). Furthermore, an analysis of the relationship between SEMA6A expression in the TMA and clinicopathological characteristics revealed that SEMA6A expression was significantly correlated with OS, tumor size, and N stage (Fig. 1e). These results suggest that SEMA6A may play a protective role in CRC. Patients were stratified into SEMA6A-high and SEMA6A-low groups based on the optimal cutoff. In GSE17538, low SEMA6A expression was associated with worse overall survival, 5-year survival, disease-specific survival, and disease-free survival (Fig. S1a), validated in our TMA cohort (Fig. S1b). Univariate and multivariate Cox regression identified SEMA6A as an independent prognostic factor (Fig. S1c-d).In the TMA, combining age, clinical stage, and SEMA6A expression yielded high predictive performance (Fig. S1e). A nomogram incorporating SEMA6A was constructed using the GSE17538 dataset, showing good calibration and high AUC (Fig. S1f-h).

To validate the inhibitory effect of SEMA6A, a stable pLV-SEMA6A cell line was established (Fig. 1f). CCK8 assay showed that SEMA6A overexpression significantly reduced cell proliferation at 24 and 48 h (Fig. 1g). Wound healing and Transwell assays demonstrated reduced migration and invasion, respectively (Fig. 1h-i). These results indicate that SEMA6A suppresses malignant progression of CRC cells.

EMT is a key process in tumor metastasis, characterized by reduced E-cadherin and increased vimentin. SEMA6A overexpression significantly reduced EMT-related markers by RT-qPCR (Fig. 1j), and immunofluorescence confirmed increased E-cadherin and decreased vimentin (Fig. 1k). Thus, SEMA6A impedes EMT in CRC.

Conversely, SEMA6A knockdown in LoVo cells reversed these phenotypes, leading to increased EMT markers (Fig. 1l), enhanced proliferation (Fig. 1m), and greater invasion (Fig. 1n). Together, these data indicate that SEMA6A suppresses CRC progression and may serve as a prognostic indicator.

### Multi-omics identifies ISG15 as a downstream oncogenic target of SEMA6A in CRC

**Fig. 2:**
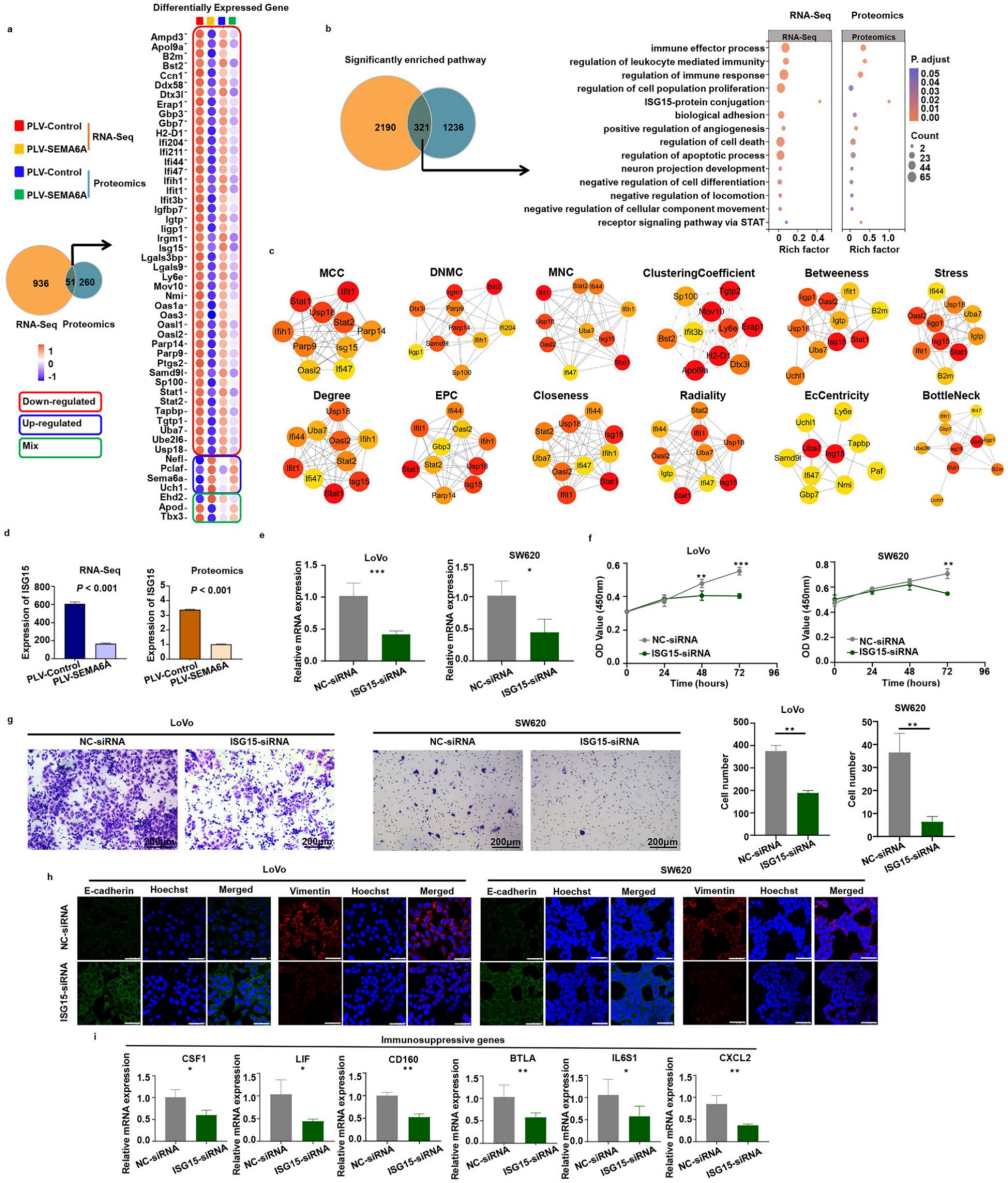
Multi-omics identification of ISG15 as a downstream target of SEMA6A and validation of its oncogenic role in CRC. **a** Transcriptome and proteome sequencing identify differentially expressed genes in SL4 and pLV-SEMA6A CRC cell lines. **b** Shared signaling pathways identified through multi-omics sequencing. **c** Identification of potential targets for SEMA6A regulation of CRC progression using 12 algorithms. **d** Transcriptome and proteome sequencing analysis of ISG15 expression in control and SEMA6A overexpressing groups. **e** RT-qPCR analysis of ISG15 knockdown efficiency in LoVo and SW620 cells. **f**-**g** Proliferation (**f**) and migration (**g**) ability of LoVo and SW620 cells after ISG15 knockdown (scale bar, 200 μm). **h** Immunofluorescence analysis of EMT-related biomarkers in LoVo and SW620 cells after ISG15 knockdown (scale bar, 50 μm). **i** RT-qPCR detection of immunosuppressive genes in control and ISG15-siRNA-transfected LoVo cells. (*P < 0.05, **P < 0.01, ***P < 0.001)

Potential regulatory genes were identified through transcriptome and proteome sequencing of SEMA6A-overexpressing SL4 cell lines. Transcriptome sequencing revealed 936 differentially expressed genes (DEGs), while proteome sequencing identified 260 DEGs. Intersection analysis highlighted 51 potential regulatory targets. Of these, 44 genes were downregulated and 4 genes were upregulated at both the transcriptome and proteome levels, as shown in the balloon plots (Fig. 2a). Pathway enrichment analyses of the differential genes revealed 2,190 enriched signaling pathways at the transcriptome level and 1,236 at the proteome level, with 321 pathways shared between the two. Immune-related pathways, along with those linked to proliferation, adhesion, migration, neovascularization, and apoptosis, were prominently enriched (Fig. 2b). To further investigate the molecular mechanisms, the 51 identified DEGs were analyzed using the Cytohubba plugin of Cytoscape software (Fig. 2c), indicated that the ISG15 gene was the most frequently identified across the 12 algorithms, suggesting its key role in the regulation of CRC malignancy by SEMA6A.

Transcriptome and proteome sequencing results revealed that the SEMA6A-high group exhibited significantly lower levels of ISG15 expression (Fig. 2d). Consistently, correlation analysis in three independent GEO datasets (GSE29621, GSE38832, and GSE31595) demonstrated a strong negative correlation between SEMA6A and ISG15 expression (Fig. S2a). To explore the impact of ISG15 on CRC prognosis, data from the GSE17538 database were analyzed. High ISG15 expression was associated with worse overall survival and 5-year survival in the GSE17538 dataset (Fig. S2b). Moreover, patients with SEMA6A^high^ISG15^low^ expression had significantly better overall survival (Fig. S2c). Thus, it is hypothesized that SEMA6A may impact CRC survival and prognosis by regulating ISG15 expression.

To validate ISG15’s role in CRC malignancy, siRNA-mediated knockdown was performed in LoVo and SW620 cells, with efficient reduction confirmed by RT-qPCR (Fig. 2e). ISG15 knockdown significantly suppressed proliferation (CCK8, Fig. 2f), migration (Transwell, Fig. 2g), and induced E-cadherin upregulation and vimentin downregulation (immunofluorescence, Fig. 2h). Given the enrichment of immune-related pathways in multi-omics, we examined immunosuppressive factors by RT-qPCR; ISG15 knockdown significantly downregulated these factors (Fig. 2i).

### SEMA6A suppresses ISG15 expression via inhibiting STAT3 signaling in CRC

**Fig. 3:**
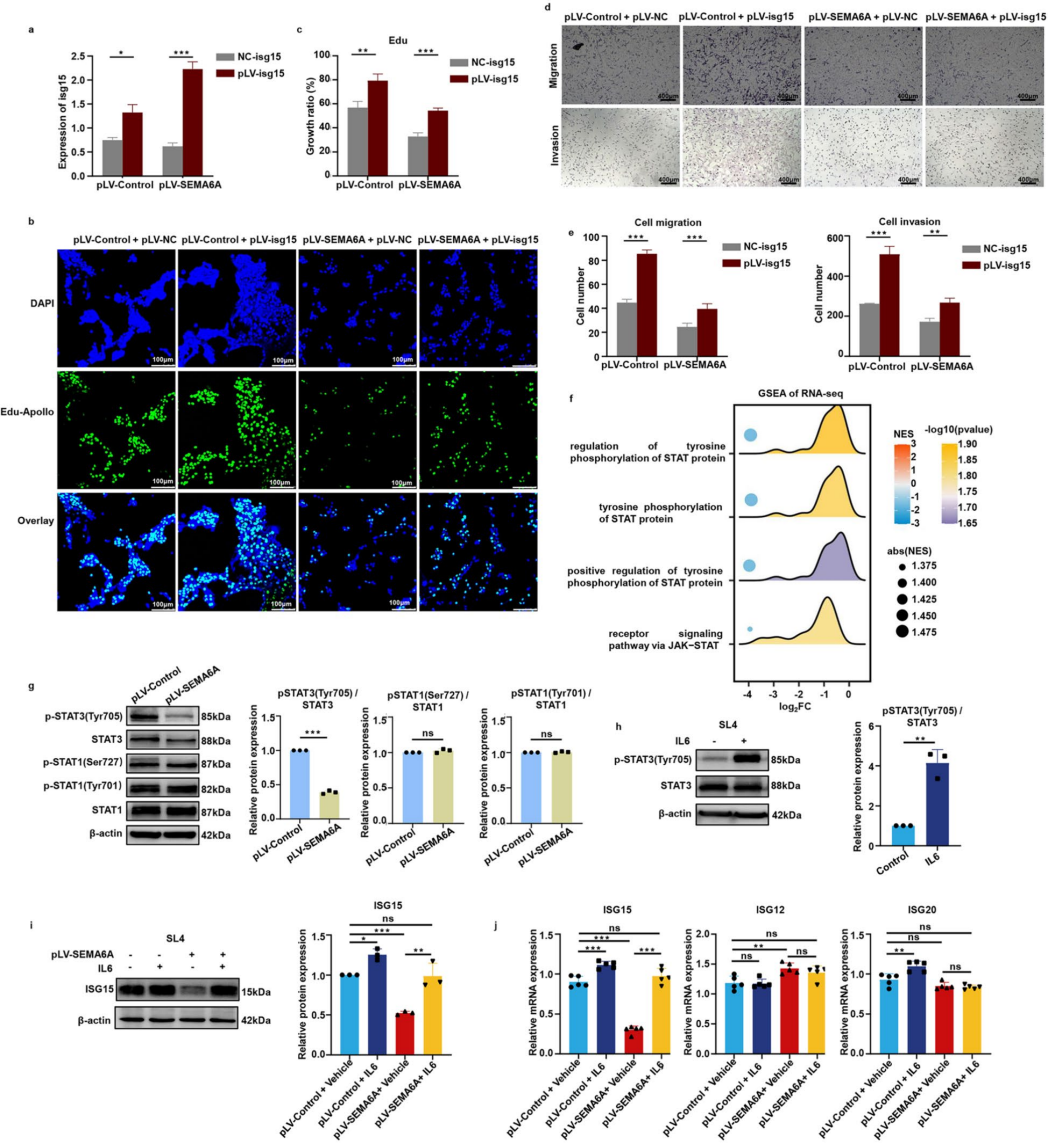
SEMA6A regulates CRC cell proliferation, migration and invasion through the STAT3/ISG15 axis. **a** RT-qPCR analysis of ISG15 overexpression efficiency in pLV-Control and pLV-SEMA6A cells. **b**-**c** EdU assay to assess the proliferation of control and pLV-ISG15 SL4 cells (scale bar, 100 μm). **d**-**e** Migration and invasion ability of control and pLV-ISG15 SL4 cells (scale bar, 400 μm). **f** GSEA showing enrichment of the JAK-STAT signaling pathway in pLV-Control group. **g** Western blot analysis of p-STAT3(Tyr705), STAT3, p-STAT1(Ser727), p-STAT1(Tyr701), and STAT1 protein levels in pLV-Control and pLV-SEMA6A cells. **h** Western blot analysis of p-STAT3(Tyr705) and STAT3 protein levels in SL4 cells following IL-6 stimulation (50ng/mL) for 6h. **i** Western blot analysis of ISG15 protein levels in pLV-Control and pLV-SEMA6A cells following IL-6 stimulation (50ng/mL) for 6h. **j** RT-qPCR analysis of ISG15, ISG12, and ISG20 mRNA expression in pLV-Control and pLV-SEMA6A cells following IL-6 stimulation (50ng/mL) for 6 h.(*P < 0.05, **P < 0.01, ***P < 0.001)

To investigate whether ISG15 mediates the functional effects of SEMA6A, we overexpressed ISG15 in pLV-Control and pLV-SEMA6A SL4 cells. RT-qPCR confirmed successful ISG15 overexpression (Fig. 3a). EdU assay showed that ISG15 overexpression significantly increased the proliferation of both pLV-Control and pLV-SEMA6A cells (Fig. 3b-c). Similarly, Transwell migration and invasion assays revealed that ISG15 overexpression enhanced the migratory and invasive capacities of these cells (Fig. 3d-e). These results indicate that ISG15 counteracts the SEMA6A-mediated suppression of CRC cell malignant phenotypes.

To explore the underlying signaling pathway, we performed GSEA on RNA-seq data from pL-Control and pLV-SEMA6A cells. The JAK-STAT signaling pathway was significantly enriched in the pLV-Control group, suggesting that SEMA6A overexpression suppresses this pathway (Fig. 3f). Western blot analysis further demonstrated that SEMA6A overexpression reduced the phosphorylation of STAT3 at Tyr705, but did not affect the phosphorylation of STAT1 at Ser727 or Tyr701 (Fig. 3g). To confirm that STAT3 can be activated in SL4 cells, we treated the cells with IL-6 (50 ng/mL) for 6 h. IL-6 stimulation markedly increased p-STAT3 (Tyr705) levels (Fig. 3h). Notably, IL-6 also induced ISG15 protein expression, and this induction was significantly attenuated in pL-SEMA6A cells compared with pLV-Control cells (Fig. 3i). Furthermore, RT-qPCR analysis showed that IL-6 increased the mRNA levels of ISG15 and ISG20, but did not affect ISG12. SEMA6A overexpression specifically suppressed the IL-6-induced upregulation of ISG15, without affecting the expression of ISG12 or ISG20 (Fig. 3j). Collectively, these data demonstrate that SEMA6A specifically downregulates ISG15 expression through inhibiting STAT3 signaling, with no effect on other ISG family members.

### In vivo experiments demonstrated the protective role of SEMA6A in CRC

**Fig. 4:**
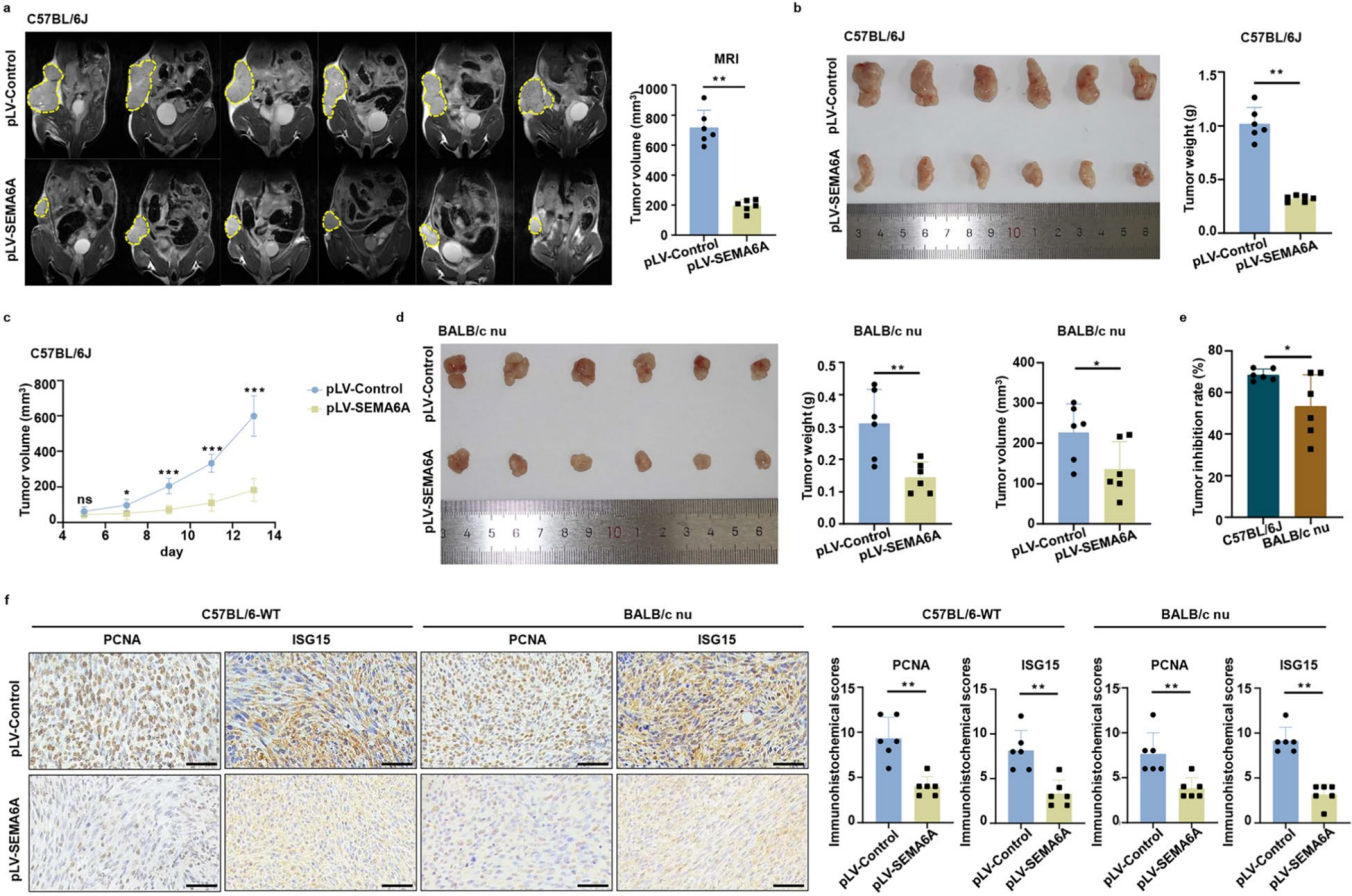
In vivo validation of the inhibitory effect of SEMA6A in CRC. **a** Magnetic resonance imaging assessment of C57BL/6J mice with subcutaneous tumors. Subcutaneous tumors are outlined by yellow dashed lines. **b** Representative images and tumor weight of subcutaneous tumors from C57BL/6J mice inoculated with pLV-Control or pLV-SEMA6A cells. **c** Tumor volume measurements in C57BL/6J mice after subcutaneous inoculation of pLV-Control or pLV-SEMA6A cells. **d** Representative images, weight, and volume of subcutaneous tumors from BALB/c nu mice inoculated with pLV-Control or pLV-SEMA6A cells. **e** Comparison of tumor inhibition rates between C57BL/6J and BALB/c nu mice. **f** Immunohistochemical staining to detect protein expression levels of PCNA and ISG15 in tumor tissues (scale bar, 50 μm). (*P < 0.05, **P < 0.01, ***P < 0.001)

Subcutaneous tumor models in both immunocompetent and immunodeficient mice were employed to further investigate the role of SEMA6A in CRC development. Magnetic resonance imaging (MRI) on day 14 in C57BL/6J mice showed smaller tumor volumes in the pLV-SEMA6A group (Fig. 4a), with lower tumor weight (Fig. 4b). Tumor volume measurements from day 5 to day 13 revealed significant differences from day 7 onward, with slower growth in the pLV-SEMA6A group (Fig. 4c). In BALB/c nu mice, SEMA6A overexpression also reduced tumor weight and volume (Fig. 4d). Tumor inhibition rates were higher in immunocompetent mice (Fig. 4e), indicating an immune-mediated component, while SEMA6A still suppressed tumor growth in nude mice, suggesting additional immune-independent mechanisms.

The proliferative capacity of tumor cells is a key indicator of malignancy, with PCNA serving as a specific biomarker for tumor proliferation. IHC staining of PCNA in tumor tissues showed a notable downregulation in subcutaneous tumors derived from both C57BL/6 J and BALB/c nu mice overexpressing SEMA6A. Additionally, this study examined the expression of ISG15, a downstream molecule of SEMA6A, and observed a similar reduction (Fig. 4f). These results further suggest that SEMA6A overexpression inhibits tumor growth by suppressing ISG15 expression.

To determine whether immune activation precedes tumor shrinkage, we analyzed tumor tissues at an early time point (day 5 post-inoculation), when tumor volumes were comparable between the pLV-Control and pLV-SEMA6A groups. Immunohistochemical staining revealed that SEMA6A overexpression already significantly increased the infiltration of CD8^+^ T cells and upregulated the effector molecules GZMA, PRF1, and IFN-γ at this early stage (Fig. S3). These findings indicate that SEMA6A promotes CD8^+^ T cell-mediated immunity before detectable tumor growth inhibition, supporting a causal role of immune activation rather than a secondary consequence of tumor reduction.

### SEMA6A-ISG15 axis modulates CD8^+^ T cell function

**Fig. 5:**
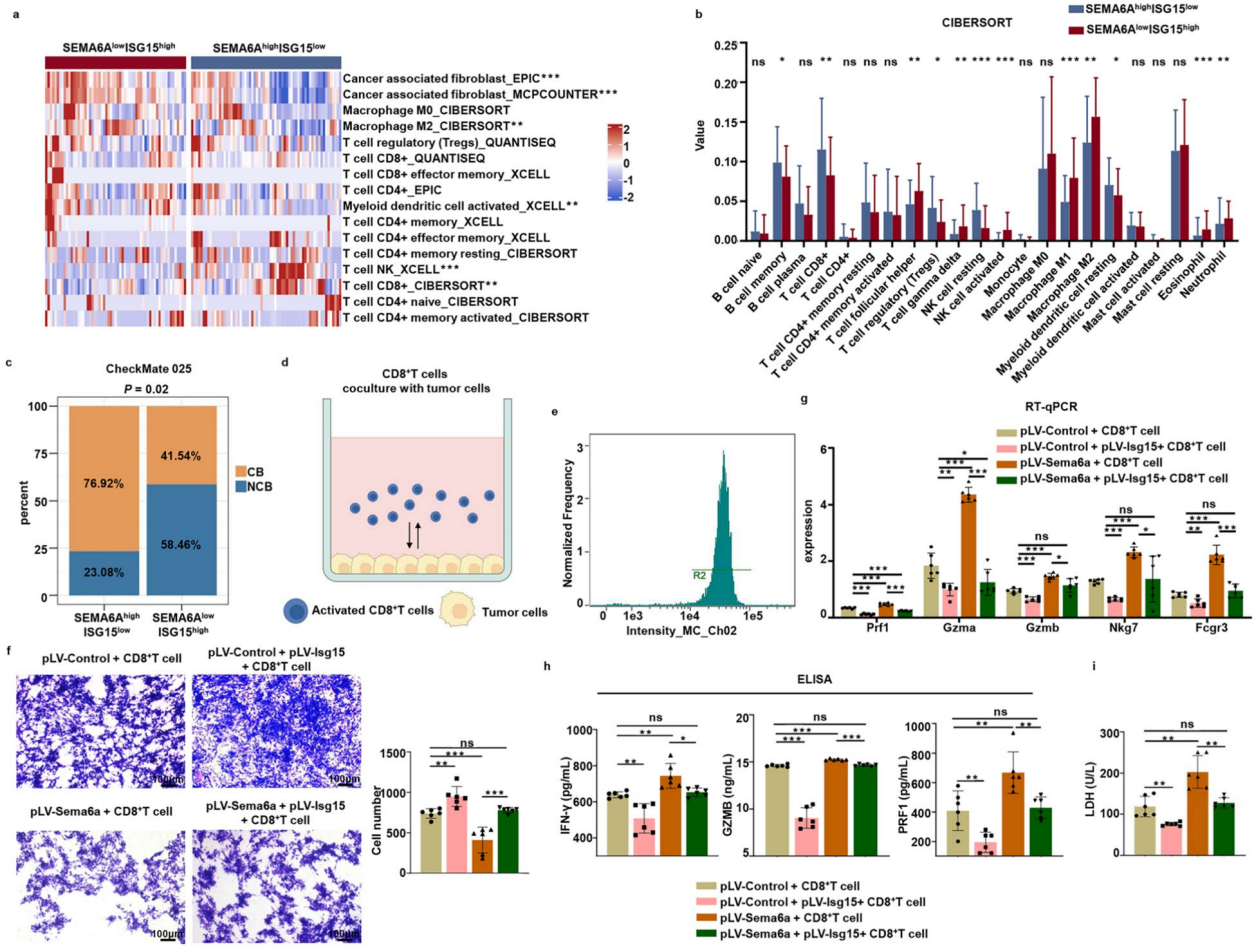
Co-culture experiments to validate the effect of SEMA6A and ISG15 on the immune system. **a** Heat map of immune cell infiltration predicted by TIMER3.0 database for patients in the GSE17538 dataset. **b** Boxplot of immune cell infiltration calculated by CIBERSORT algorithm. **c** Validation of immunotherapy predictability using the CheckMate 025 dataset in relation to SEMA6A and ISG15 expression. **d** Co-culture setup of activated CD8^+^ T cells and SL4 cells. **e** Flow cytometry plot showing CD8^+^ T cell purity after isolation. **f** Crystal violet staining results following co-culture of SL4 cells with CD8^+^ T cells, with or without SEMA6A and ISG15 overexpression (scale bar, 200 μm). **g** RT-qPCR analysis of mRNA expression of cytotoxicity-related genes. **h** Quantitative analysis of effector cytokine IFN-γ, GZMB, and PRF1 from activated CD8^+^ T cells, detected by ELISA. **i** LDH cytotoxicity assay to measure the cytotoxic effects of CD8.^+^ T cells. (*P < 0.05, **P < 0.01, ***P < 0.001)

Given that SEMA6A overexpression exhibited stronger tumor inhibition in immunocompetent mice than in immunodeficient mice (Fig. 4e), we explored whether SEMA6A modulates immune cell function through the ISG15 axis. Patients from the GSE17538 dataset were stratified into SEMA6A^high^ISG15^low^ and SEMA6A^low^ISG15^high^ groups. Using the TIMER3.0 database, we analyzed immune cell infiltration across multiple algorithms (Fig. 5a). Notably, the CIBERSORT algorithm revealed significantly higher CD8⁺ T cell infiltration in the SEMA6A^high^ISG15^low^ group, with no difference for CD4⁺ T cells (Fig. 5b). Analysis of the CheckMate 025 dataset further indicated that this group derived greater benefit from immunotherapy (Fig. 5c).

To validate the functional impact of this axis, we established a co-culture system of activated CD8^+^ T cells and SL4 CRC cells (Fig. 5d). Flow cytometry confirmed that over 90% of isolated cells were CD8^+^ T cells (Fig. 5e). Crystal violet staining showed that SEMA6A overexpression significantly reduced tumor cell viability, whereas ISG15 overexpression reversed this effect (Fig. 5f). RT-qPCR analysis of co-culture supernatants revealed that SEMA6A overexpression increased the mRNA levels of cytotoxicity-related genes, while ISG15 overexpression decreased them (Fig. 5g). Enzyme-linked immunosorbent assay (ELISA) quantification of IFN-γ, GZMB, and PRF1 (Fig. 5h) and LDH cytotoxicity assay (Fig. 5i) further confirmed that SEMA6A enhanced CD8^+^ T cell-mediated killing, whereas ISG15 suppressed it. Collectively, these data demonstrate that the SEMA6A-ISG15 axis specifically regulates CD8^+^ T cell effector function in CRC.

### ISG15 stabilizes TGF-β1 via ISGylation to promote CRC progression

**Fig. 6:**
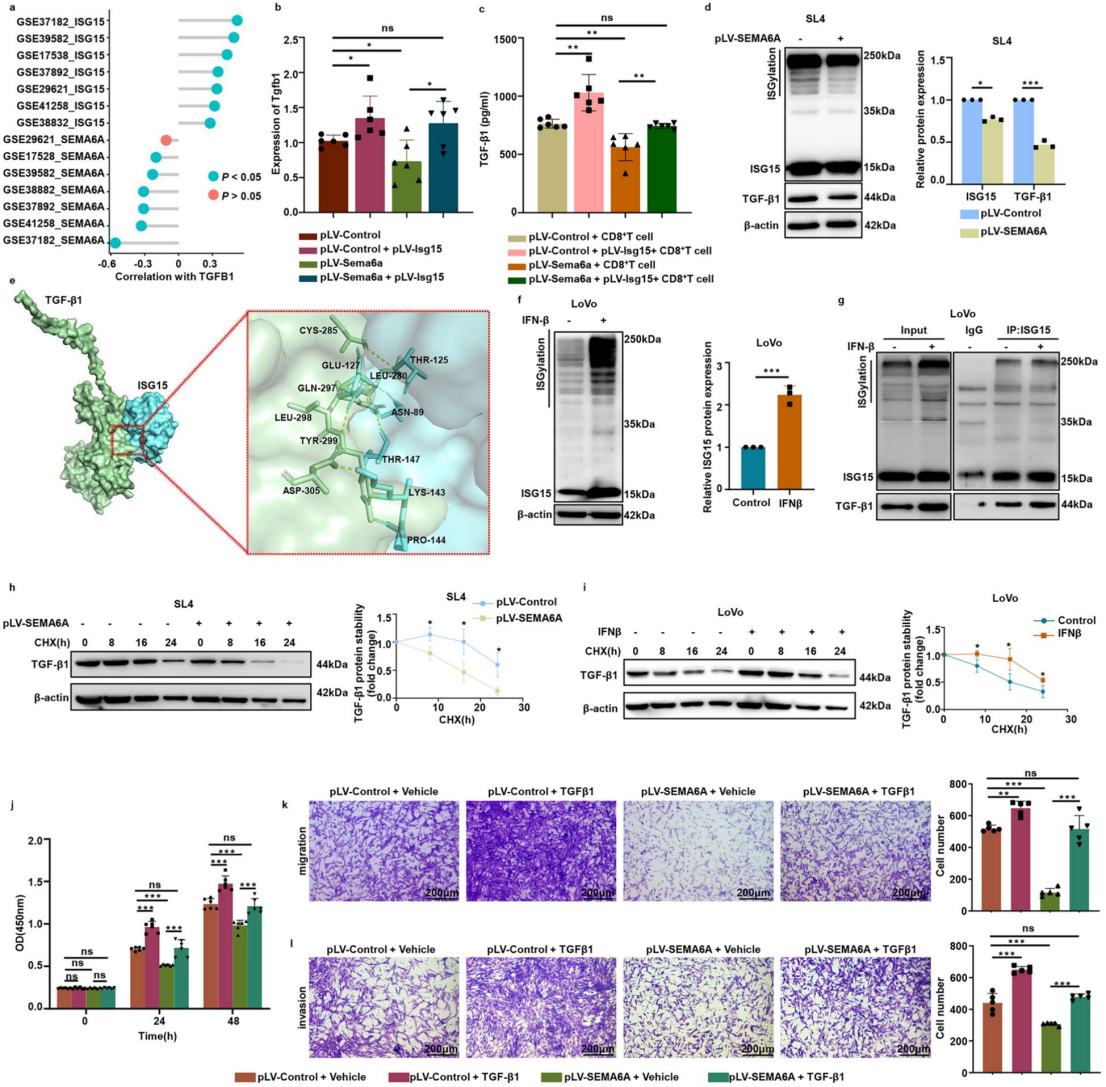
ISG15 stabilizes TGF-β1 protein through ISGylation to regulate CRC progression. **a** Lollipop plot showing correlations of SEMA6A, ISG15, and TGFB1 expression across GEO datasets. **b** RT-qPCR analysis of TGFB1 mRNA expression in CD8^+^ T cells from co-culture systems. **c** ELISA detection of TGF-β1 secretion in the supernatant of co-culture systems. **d** Western blot analysis of ISGylation, ISG15, and TGF-β1 protein levels in pLV-Control and pLV-SEMA6A cells. **e** Molecular docking visualization of the interaction between TGF-β1 and ISG15. **f** Western blot analysis of ISGylation and ISG15 protein levels in LoVo cells following IFN-β stimulation (20ng/mL) for 24h. **g** Co-immunoprecipitation assay detecting the interaction between ISG15 and TGF-β1 (immunoprecipitation with anti-ISG15 antibody). **h** Western blot analysis of TGF-β1 protein stability in pLV-Control and pLV-SEMA6A cells treated with CHX for 0, 8, 16, and 24h. **i** Western blot analysis of TGF-β1 protein stability in LoVo cells treated with IFN-β (20ng/mL) followed by CHX for 0, 8, 16, and 24h. **j**-**l** After TGF-β1 supplementation (10ng/mL) in pLV-Control and pLV-SEMA6A cells, cell proliferation (**j**) was assessed by CCK8 assay, and cell migration (**k**) and invasion (**l**) were evaluated by Transwell assay (scale bar: 200 μm). (*P < 0.05, **P < 0.01, ***P < 0.001)

Given the impact of SEMA6A and ISG15 on tumor cell progression in cellular and nude mouse models, their effects on tumor cells were further investigated. Preliminary cellular experiments revealed that SEMA6A overexpression and ISG15 knockdown impeded the EMT process in CRC cells. TGF-β1, a key EMT promoter via SMAD signaling, plays a critical role in immune evasion [21]. To explore the relationship between TGF-β1 and the SEMA6A-ISG15 axis, multiple datasets were analyzed, revealing that TGFB1 expression negatively correlated with SEMA6A and positively with ISG15 (Fig. 6a). RT-qPCR analysis of TGFB1 expression in CRC cells revealed that TGF-β1 levels were notably reduced in cells overexpressing SEMA6A and significantly increased in cells overexpressing ISG15 (Fig. 6b). ELISA assays of TGF-β1 concentrations in co-culture supernatants confirmed these findings (Fig. 6c).

Given that our multi-omics analysis significantly enriched the ISG15-protein conjugation pathway, we next examined whether ISG15 regulates TGF-β1 through ISGylation. Western blot analysis revealed that SEMA6A overexpression reduced global ISGylation, ISG15 protein, and TGF-β1 levels in SL4 cells (Fig. 6d). To predict whether ISG15 directly binds to TGF-β1, molecular docking was performed, which suggested a potential interaction (Fig. 6e). To experimentally validate this interaction, we first induced ISGylation in LoVo cells by stimulating with IFN-β (20 ng/mL for 24 h), which markedly elevated ISGylation and ISG15 expression (Fig. 6f). Under this condition, co-immunoprecipitation using an anti-ISG15 antibody confirmed that ISG15 physically interacts with TGF-β1 (Fig. 6g), providing evidence that TGF-β1 is a substrate for ISGylation. To determine whether ISGylation affects TGF-β1 protein stability, cycloheximide (CHX) chase assays were performed. In pLV-SEMA6A cells, TGF-β1 degraded more rapidly than in control cells, indicating that SEMA6A overexpression (which suppresses ISGylation) destabilizes TGF-β1 (Fig. 6h). Conversely, IFN-β pretreatment, which upregulates ISG15 and ISGylation, prolonged the half-life of TGF-β1 in LoVo cells (Fig. 6i). Functionally, adding exogenous TGF-β1 (10 ng/mL) rescued the proliferation, migration, and invasion defects caused by SEMA6A overexpression in SL4 cells (Fig. 6j-l). Collectively, these data demonstrate that ISG15 stabilizes TGF-β1 through ISGylation, thereby promoting CRC progression.

### SEMA6A overexpression enhances the anti-tumor efficacy of PD-1 blockade in vivo

**Fig. 7:**
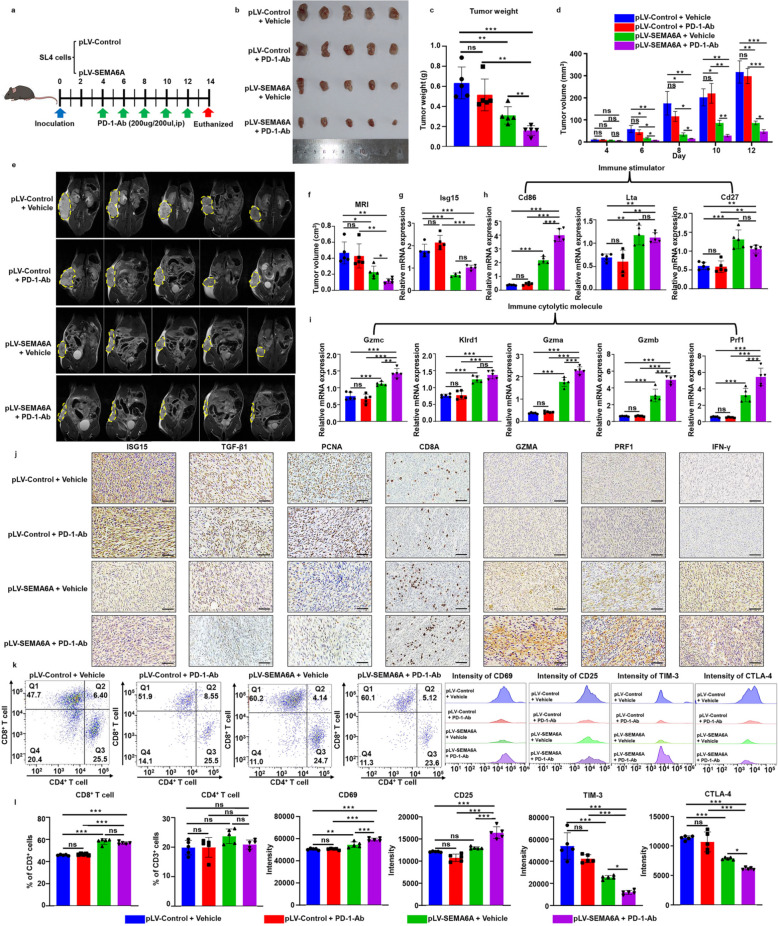
SEMA6A overexpression enhances the anti-tumor efficacy of PD-1 blockade in vivo. **a** Schematic representation of a subcutaneous tumor model in C57BL/6J mice. **b** Representative images of subcutaneous CRC tumor models. **c**-**d** Tumor weight (**c**) and tumor volume (**d**) analyzed by one-way ANOVA in four groups. **e**–**f** Magnetic resonance imaging assessment of C57BL/6J mice with subcutaneous tumors, tumors are outlined by yellow dashed lines. **g**-**i** RT-qPCR analysis of specific biomarker mRNA expression in tumors from the four treatment groups. **j** Immunohistochemical staining of ISG15, TGF-β1, PCNA, CD8A, GZMA, PRF1, IFN-γ in tumor tissues (scale bar, 50 μm). **k**-**l** Flow cytometry analysis of CD8^+^ T cell and CD4^+^ T cell infiltration, as well as the mean fluorescence intensity of CD69, CD25, TIM-3, and CTLA-4 in tumor tissues. (*P < 0.05, **P < 0.01, **P < 0.001)

Bioinformatics analysis and experimental validation revealed that SEMA6A influences the response of CRC patients to immunotherapy. To further assess its role, a mouse subcutaneous tumor model was established (Fig. 7a). Representative tumor images (Fig. 7b) and quantitative analysis showed that anti-PD-1 monotherapy did not significantly reduce tumor weight or volume compared to the control group. In contrast, SEMA6A overexpression alone significantly suppressed tumor growth, and the combination of SEMA6A overexpression with anti-PD-1 further reduced tumor weight and volume to the greatest extent (Fig. 7c-d). MRI confirmed these findings (Fig. 7e-f). To explore the underlying mechanisms, we examined the expression of immune-related biomarkers in tumor tissues. RT-qPCR analysis showed that ISG15 mRNA expression was significantly reduced in both the pLV-SEMA6A + Vehicle group and the pLV-SEMA6A + PD-1-Ab group compared with the control group (Fig. 7g). Meanwhile, the combination treatment most effectively upregulated the immunostimulatory factors CD86, LTA, and CD27 (Fig. 7h), as well as the cytotoxic effector molecules (Fig. 7i). IHC staining (Fig. 7j) and quantitative analysis (Fig. S4) were performed on tumor tissues from the four treatment groups. The two control groups (pLV-Control + Vehicle and pLV-Control + PD-1-Ab) showed no significant differences in any marker. Compared with controls, SEMA6A overexpression alone (pLV-SEMA6A + Vehicle) reduced ISG15, TGF-β1, and PCNA levels, while increasing CD8A, GZMA, PRF1, and IFN-γ. Addition of anti-PD-1 to SEMA6A overexpression further lowered TGF-β1 and PCNA and further elevated GZMA and PRF1, but did not significantly change ISG15, CD8A, or IFN-γ compared with SEMA6A alone. These data indicate that SEMA6A alone suppresses tumor-promoting factors and enhances CD8^+^ T cell-mediated immunity, and that PD-1 blockade provides additional benefit in specific cytotoxic effectors and proliferation control. Flow cytometry further demonstrated that the combination treatment significantly increased the infiltration of CD8^+^ T cells (but not CD4^+^ T cells) and upregulated the activation markers CD69 and CD25, while decreasing the exhaustion markers TIM-3 and CTLA-4 (Fig. 7k-l). Collectively, these data demonstrate that SEMA6A overexpression sensitizes CRC tumors to PD-1 blockade by enhancing CD8^+^ T cell infiltration and effector function.

## Discussion

CRC is the third most commonly diagnosed malignancy worldwide. Tumor heterogeneity complicates early diagnosis, often leading to detection at advanced stages. Current diagnostic methods, such as endoscopic and fecal blood tests, exhibit limited accuracy, contributing to delayed diagnosis. In contrast, molecular testing kits offer higher sensitivity and precision, improving diagnostic outcomes. Therefore, the development of DNA-, RNA-, and protein-based biomarkers is essential to enhance OS rates in CRC patients and advance precision oncology [22].

This study established the diagnostic and prognostic significance of SEMA6A in CRC. Integrated analysis of NanoString data and GEO validation cohorts consistently showed downregulation of SEMA6A in CRC tissues compared to normal controls. Using an optimal expression cutoff, patients stratified into SEMA6A-high and SEMA6A-low groups demonstrated significantly prolonged OS in the SEMA6A-high cohort. Multivariable Cox regression analysis confirmed SEMA6A as an independent prognostic factor, with ROC analysis and nomogram validation further demonstrating high predictive accuracy and sensitivity. These findings were supported by IHC validation and in vitro experiments, including SEMA6A overexpression and knockdown, highlighting SEMA6A as a promising prognostic indicator for clinical diagnosis and prognostic stratification in CRC.

Multi-omics analysis implicated SEMA6A in CRC progression through modulation of immune-related pathways, including JAK-STAT and ISG15-protein conjugation. Using 12 algorithms in cytoHubba, ISG15 was identified as a critical downstream effector regulated by SEMA6A. ISG15, a 15-kDa interferon-induced ubiquitin-like protein, modulates immune responses via interferon signaling [23–25]. It conjugates to target proteins through ISGylation [26–28], with both conjugated and free forms exhibiting context-dependent pro- and anti-tumor functions [29–32]. Our multi-omics data showed significant downregulation of ISG15 in the SEMA6A-high group, with elevated ISG15 expression correlating with poor CRC prognosis. Cellular functional assays confirmed ISG15's role in promoting CRC progression. Mechanistically, we found that SEMA6A inhibits the JAK-STAT3 pathway, leading to reduced ISG15 expression. ISG15 in turn stabilizes TGF-β1 through ISGylation, as evidenced by molecular docking, co-immunoprecipitation, and cycloheximide chase assays. Consequently, SEMA6A overexpression decreases TGF-β1 secretion and relieves its suppressive effect on CD8^+^ T cells. Collectively, these findings delineate a novel SEMA6A/STAT3/ISG15/TGF-β1 axis in CRC.

Consistent with in vitro results, SEMA6A overexpression significantly suppressed tumor growth in both C57BL/6J and BALB/c nu mouse models. The observation that SEMA6A inhibited tumor growth even in immunodeficient nude mice demonstrates that SEMA6A possesses intrinsic, cell-autonomous anti-tumor activity independent of adaptive immunity. Meanwhile, the tumor inhibition rate was significantly higher in immunocompetent C57BL/6J mice than in nude mice, indicating that the adaptive immune system contributes additional anti-tumor effects. To further establish causality, we analyzed tumor tissues at an early time point (day 5 post-inoculation) when tumor volumes were comparable between groups. SEMA6A overexpression already significantly increased CD8^+^ T cell infiltration and upregulated effector molecules GZMA, PRF1, and IFN-γ, indicating that immune activation precedes tumor shrinkage. These findings support that SEMA6A directly modulates the immune microenvironment as an early event rather than as a secondary consequence of tumor reduction. Collectively, our data indicate that SEMA6A exerts a dual mechanism: a direct suppression of tumor cell malignancy and an indirect enhancement of anti-tumor immunity. The relative contribution of each pathway cannot be precisely quantified from the current data, but the higher inhibition rate in immunocompetent hosts highlights the meaningful role of immune-mediated effects in complementing the intrinsic activity of SEMA6A.

Emerging evidence suggests that converting immune-desert (“cold”) tumors into immune-inflamed (“hot”) phenotypes by increasing cytotoxic T lymphocyte and NK cell infiltration can restore patient responsiveness to ICIs [33–35]. These conversion strategies may synergize with ICIs to enhance antitumor immunity and improve clinical outcomes. Our study demonstrates that SEMA6A increases the secretion of cytotoxic factors by CD8^+^ T cells in co-culture systems, as shown by increased secretion of IFN-γ, GZMB, and PRF1. Flow cytometry analysis of tumor tissues from treated mice further revealed that SEMA6A overexpression elevated the proportion of CD8^+^ T cells, upregulated activation markers (CD69, CD25), and downregulated exhaustion markers (CTLA-4, TIM-3). These results demonstrate that SEMA6A not only promotes CD8^+^ T cell infiltration but also improves their functional state.

Given that TGF-β1 is a well-established master regulator of CD8^+^ T cell dysfunction [36], our integrated bioinformatics and experimental validation confirmed that SEMA6A suppresses TGF-β1 expression via the ISG15-dependent pathway, thereby inhibiting tumor immune evasion. Critically, in subcutaneous tumor models, the combination of SEMA6A overexpression and PD-1 monoclonal antibody treatment resulted in significantly reduced tumor size and weight compared to monotherapy. This synergistic effect indicates that SEMA6A facilitates the conversion of the TME to one more responsive to ICIs. Mechanistically, the combination therapy upregulated immunostimulatory factors and enhanced cytotoxic effector functions, collectively potentiating antitumor immunity.

Despite the comprehensive findings, several limitations of this study should be acknowledged. First, the clinical data used for prognostic analysis were derived from retrospective cohorts and a tissue microarray, which may introduce inherent selection bias. Prospective multicenter studies with larger sample sizes are warranted to validate the prognostic value of SEMA6A. Second, due to the retrospective nature of the study, patient serum samples were not available, precluding assessment of circulating SEMA6A or ISG15 as potential non-invasive indicators. Third, although we demonstrated that ISG15 stabilizes TGF-β1 via ISGylation using co-immunoprecipitation and cycloheximide chase assays, direct evidence of the specific lysine residues on TGF-β1 undergoing ISGylation remains to be determined. Fourth, while our study establishes an indirect regulatory axis from SEMA6A to CD8^+^ T cells via ISG15/TGF-β1, we acknowledge that extracellular ISG15 may directly affect T cell function. Future studies using recombinant ISG15 or conditioned medium are needed to explore this possibility. Finally, the synergistic effect of SEMA6A overexpression with PD-1 blockade was observed in a subcutaneous syngeneic mouse model; further studies using orthotopic or genetically engineered mouse models are needed to better recapitulate the clinical setting and to evaluate potential adverse effects before clinical translation.

## Conclusion

**Fig. 8:**
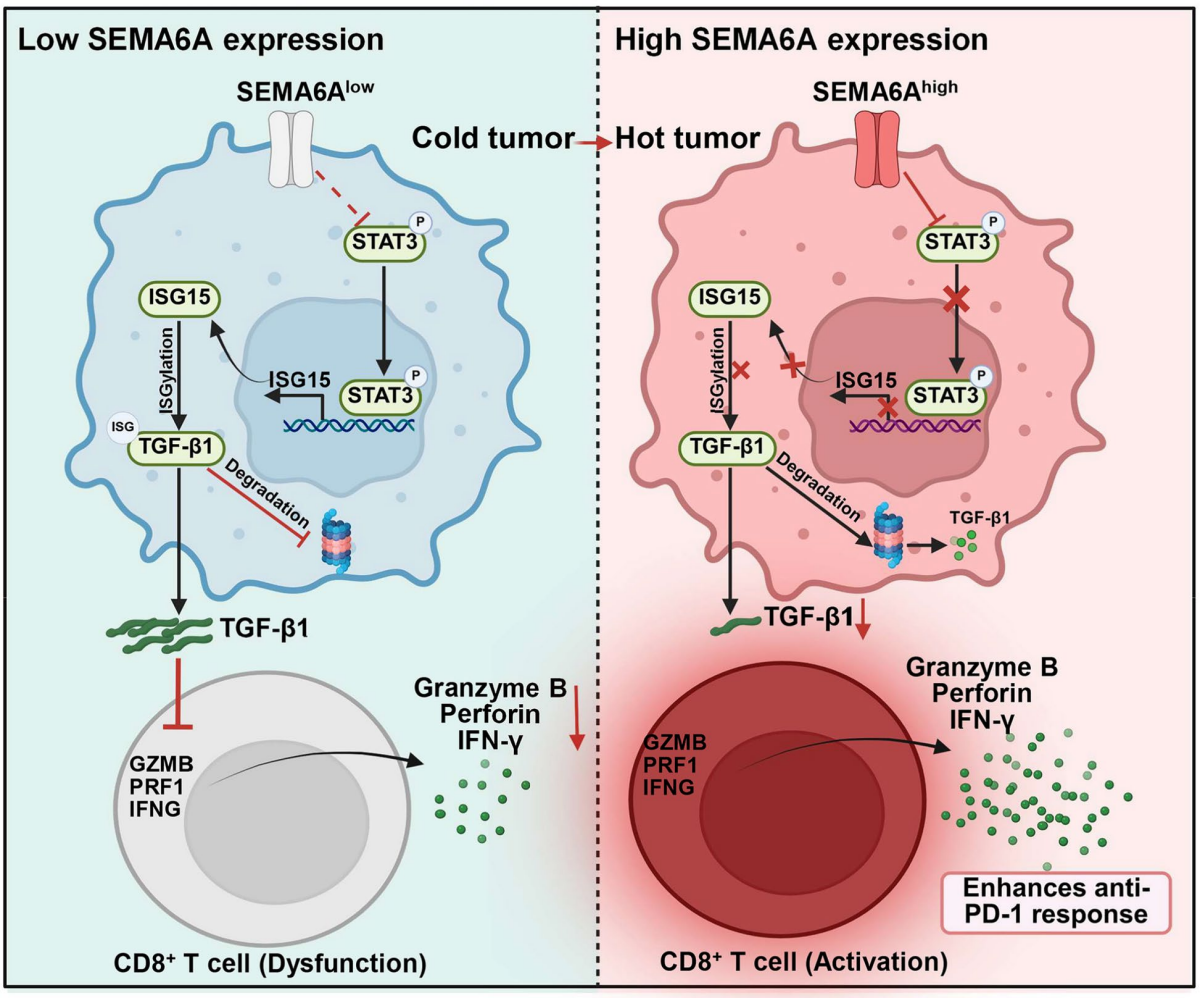
Schematic model of SEMA6A-ISG15-TGF-β1 axis in colorectal cancer. Left panel: SEMA6A downregulation leads to activation of the JAK/STAT3 pathway. Phosphorylated STAT3 translocates to the nucleus and promotes transcription of ISG15. Elevated ISG15 protein ISGylates TGF-β1, preventing its proteasomal degradation. Secreted TGF-β1 suppresses CD8^+^ T cell cytotoxicity by downregulating PRF1, GZMB, and IFN-γ expression, thereby promoting tumor immune evasion. Right panel: SEMA6A overexpression inhibits the JAK/STAT3 pathway, reducing ISG15 expression. Consequently, TGF-β1 undergoes degradation. The reduced TGF-β1 release restores CD8^+^ T cell-mediated antitumor immunity. Created with BioRender

In conclusion, integrated bioinformatics and experimental validation identified SEMA6A as a clinically significant prognostic factor in CRC. Elevated SEMA6A expression correlated with a favorable prognosis, enhanced immune activation, and served as an independent prognostic factor, highlighting its tumor-protective role. Functionally, SEMA6A suppressed malignant progression by inhibiting the STAT3/ISG15/TGF-β1 axis. Co-culture experiments demonstrated that SEMA6A enhances CD8^+^ T cell cytotoxicity by suppressing the ISG15/TGF-β1 axis, leading to increased activation and reduced exhaustion. Notably, SEMA6A overexpression synergized with PD-1 blockade in vivo, potentiating sensitivity to immune checkpoint inhibitors. Collectively, these findings suggest that targeting the SEMA6A/ISG15/TGF-β1 axis represents a dual therapeutic strategy that both directly suppresses CRC progression and enhances anti-tumor immunity, providing a rationale for combining SEMA6A modulation with immunotherapy in CRC. Based on these findings, we propose a working model illustrating how SEMA6A suppresses CRC progression and enhances anti-tumor immunity through the ISG15/TGF-β1 axis (Fig. 8).

## Materials and methods

Detailed methods for bioinformatics analyses and selected experimental procedures are available in the Supplementary Material. All of the animal protocols complied with the guidelines from EU Directive 2010/63/EU of the European Parliament on the protection of animals used for scientific purposes. The protocols were also approved by the Institutional Animal Care and Use Committee of the Institute of Materia Medica, Chinese Academy of Medical Sciences and Peking Union Medical College (Approval no. IMM-N-25–0216). The studies involving human participants were reviewed and approved by the Research Ethics Committee of The Affiliated Hospital of Qingdao University (ethics approval number: QDU-HEC-2023177). Informed consents were obtained from all of the participating patients. The studies were conducted in accordance with the local legislation and institutional requirements.

### NanoString-based RNA assay

Gene expression profiles of colon cancer tissues were analyzed using NanoString technology [37]. NanoString gene expression analysis included 12 normal colon tissue samples and 14 colorectal cancer tissue samples. No technical replicates were included in this experiment. The nCounter molecular barcodes included 256 target genes and 6 housekeeping genes. Total RNA was extracted from matched normal and colon cancer tissues, with raw data being normalized. Gene expression levels were visualized using the “ComplexHeatmap” R package [38]. Differential expression of target genes between normal and tumor tissues was assessed using the “limma” R package [39].

### Immunochemistry staining

A TMA of 90 paired CRC and normal tissues was constructed. For IHC, deparaffinized and rehydrated tissue sections were treated with 3% H₂O₂, followed by antigen retrieval in citrate buffer (100°C). After blocking with 5% BSA, sections were incubated with primary antibodies (Table S1) overnight at 4 °C, then with secondary antibodies (BST19013894, BST20023895, Boste) for 1 h at room temperature. Staining was visualized with DAB and counterstained with hematoxylin. Two blinded pathologists scored each section using the H-score method: intensity (0–3) × percentage of positive cells (0–4, where 0:0%, 1:1–25%, 2:26–50%, 3:51–75%, 4:76–100%).

### Western blot analysis

Total protein was extracted from CRC cells using lysis buffer (R0020, Solarbio) containing a protease inhibitor cocktail (C0001, TargetMol). For detection of phosphorylated proteins, a phosphatase inhibitor cocktail (SJ-MK0002-A, SparkJade) was added to the lysis buffer. For ISGylation assays, the lysis buffer was supplemented with 20 mM N-ethylmaleimide (HY-D0843, MCE) and 10 μM MG132 (HY-13259, MCE) to preserve ISGylated conjugates. Protein concentration was determined by the BCA assay (23225, ThermoFisher). Equal amounts of protein were separated by 10% SDS-PAGE and transferred to nitrocellulose membranes. After blocking, membranes were incubated with primary antibodies (Table S1) overnight at 4 °C, followed by appropriate secondary antibodies. Protein bands were visualized using an ImageQuant™ LAS 4000 luminometer (GE).

### Cell culture and transfection

Human CRC cell lines LoVo and SW620 were obtained from BNBIO (Beijing, China). The luciferase-expressing mouse CRC cell line SL4 was provided by our laboratory. Lentivirus for SEMA6A overexpression (pLV-SEMA6A), siRNAs targeting human SEMA6A and ISG15, and lentivirus for mouse Isg15 overexpression were synthesized by Beijing Miga Technology Co. (Beijing, China). All cells were maintained at 37 °C in a 5% CO₂ incubator. Transfections were performed according to the manufacturers’ protocols.

### Proliferation, migration, and invasion assays

Cell proliferation was evaluated using CCK-8 (CT0001-A, SparkJade) and EdU (E-CK-A378, Elabscience) assays. For CCK-8, cells were seeded at 5 × 10^3^ cells/well in 96-well plates; at indicated times, 10 µL CCK-8 solution was added, incubated for 4 h, and absorbance at 450 nm measured. For EdU, cells were incubated with EdU reagent for 2 h, fixed with 4% paraformaldehyde, stained with Apollo and DAPI, and visualized by confocal microscopy (Leica SP8X, Germany).

Wound healing assays were performed using scratch inserts. Cells (1 × 10^5^/well) were seeded in 24-well plates. After insert removal, scratch closure was photographed at 0, 24, 48, and 72 h. Transwell migration and invasion assays were carried out using 8-µm inserts (Corning) uncoated or coated with Matrigel. Cells (2 × 10^4^/well) were seeded into the upper chamber with 1% FBS; the lower chamber contained 10% FBS. After 48 h, migrated/invaded cells were fixed, stained with 0.1% crystal violet, and counted.

### Immunofluorescence

Immunofluorescence staining was performed on SL4 and pLV-SEMA6A cells cultured in confocal dishes for 48 h. After fixation with 4% paraformaldehyde, cells were blocked with 5% BSA and permeabilized with 0.3% Triton X-100. Primary antibodies against vimentin (1:100; BM0135; Boster) or E-cadherin (1:50; sc-8426; Santa Cruz) were incubated with fixed cells overnight at 4 °C. The secondary antibodies (1:200; Boster) were then applied for 1 h at room temperature. The nuclei were stained with Hoechst for 10 min, and fluorescence images were captured using a confocal microscope (Leica SP8X, Germany).

### RNA extraction and RT-qPCR

Total RNA was extracted from cells or tumor tissues using an RNeasy kit (AC0202-B, SparkJade). For reverse transcription, 1 μg of total RNA was processed with SuperScript II reverse transcriptase (AG0305-B, SparkJade). Quantitative PCR analysis was performed using an ABI 7900 HT real-time PCR system and SYBR Green Master Mix (AH0104-C, SparkJade, Shandong, China). The RT-qPCR primer sequences are provided in Table S2.

### RNA-seq and proteomics analysis

The SL4 and pLV-SEMA6A cell lines were sent to Beijing YIKEBAIDE Technology Co., LTD., where RNA extraction, quality assessment, library construction, and sequencing were performed. Interaction analysis was subsequently conducted on the Beijing Meiji Biological cloud platform. Detailed procedures are provided in Supplementary Material.

### Mouse CD8⁺ T cell isolation and co-culture

CD8⁺ T cells were isolated from C57BL/6 mouse splenocytes using a CD8⁺ Cell Isolation Kit (19,853, STEMCELL). Purity was verified by flow cytometry with FITC anti-mouse CD8a antibody (100705, BioLegend). Purified CD8⁺ T cells were activated with anti-CD3e (553087, BD Biosciences), anti-CD28 (553294, BD Biosciences), and IL-2 (Eg0456, Proteintech)for 48 h, then co-cultured with mouse CRC cells at a 8:1 ratio (CD8⁺ T:CRC). After 48 h, supernatants were collected for ELISA detection of IFN-γ (EMC101g, Neobioscience), PRF1 (E-EL-M0890, Elabscience), GZMB (E-EL-M0594) and TGF-β1 (Neobioscience). Cytotoxicity was assessed by measuring LDH activity using a TBA-40FR ACCUTE fully automatic biochemical analyzer (TOSHIBA, Japan).

### Animal studies

Female 8-week-old C57BL/6J and BALB/c nu mice were purchased from HFK Bio-Technology. For subcutaneous tumor models, SL4 or pLV-SEMA6A cells (1 × 10⁶ in 100 µL saline) were injected into the left hind leg. Tumor growth was monitored in vivo using a PharmaScan 70/16 US (7.0 T, Bruker, Switzerland) MRI scanner on day 14 prior to sacrifice. Tumor volume was calculated using Radiant software. For immunotherapy experiments, anti-PD-1 monoclonal antibody (200 µg/200 µL) was intraperitoneally injected on days 4, 6, 8,10 and 12.

### Flow cytometry analysis of tumor-infiltrating lymphocytes

Tumor tissues were minced and digested with collagenase IV and DNase I for 1 h at 37 °C. Single-cell suspensions were stained with the antibodies list in Table S1. Data were acquired on a flow cytometer and analyzed with FlowJo software. The percentages of CD8⁺ and CD4⁺ T cells among total live cells, as well as the mean fluorescence intensity of activation and exhaustion markers, were calculated.

### Co-immunoprecipitation

Cells were lysed in IP lysis buffer (P0013, Beyotime) supplemented with protease inhibitor cocktail. For ISGylation detection, 20 mM N-ethylmaleimide (NEM) and 10 μM MG132 were added to the lysis buffer. Lysates were incubated with anti-ISG15 antibody (sc-166755, Santa Cruz) or control IgG (A7028, Beyotime) overnight at 4 °C, followed by incubation with protein A/G-agarose beads for 4 h. Beads were washed, and bound proteins were eluted by boiling in SDS loading buffer, separated by SDS-PAGE, and immunoblotted with anti-TGF-β1 (81,746–2-RR, Proteintech) and anti-ISG15 antibody (15,981–1-AP, Proteintech).

### Cycloheximide protein stability assay

Cells were treated with CHX (50 μg/mL, HY-12320, MCE) for 0, 8, 16, and 24 h. At each time point, cells were harvested and lysed. Equal amounts of protein were subjected to Western blotting with anti-TGF-β1 antibody. Protein band intensities were quantified and normalized to β-actin, and the remaining percentage of TGF-β1 was plotted against time.

## Supplementary Information

Supplementary Material 1.

## Data Availability

The original contributions presented in the study are included in the article and Supplementary Material, and the RNA-seq data have been deposited in the GSE298450.
