# Supplementary material for: SEMA6A inhibits tumor progression and boosts anti-tumor immunity via blocking the ISG15/TGFβ axis in colorectal cancer
Source: Mol Biomed. 2026 Jun 17;7:93. doi: 10.1186/s43556-026-00493-6 (PMC13272749; doi:10.1186/s43556-026-00493-6)
Supplement: Supplementary file 1 — Supplementary Material 1. [file 43556_2026_493_MOESM1_ESM.docx]

**Title:** SEMA6A inhibits tumor progression and boosts anti-tumor immunity *via* blocking the ISG15/TGFβ axis in colorectal cancer

**Authors**: Fang Zhang^1,2#^, Rixin Zhang^1,2#^, Zheng Yan^1,2^, Jinbao Zong^3,4^, Mingxuan Zhou^1,2^, Tiegang Li ^1,2^, Yufang Hou^1,2^, Silin Lv^1,2^, Zifan Zeng^1,2^, Wenyi Zhao^1,2^, Yixin Zhou^1,2^, Zengni Zhu^1,2^, Siying Huang^1,2^, Min Yang^1,2*^

**# These authors contributed equally.**

**Affiliations:** ^1^ State Key Laboratory of Digestive Health, Institute of Materia Medica, Chinese Academy of Medical Sciences and Peking Union Medical College, Beijing 100050, China. ^2^ State Key Laboratory of Bioactive Substance and Function of Natural Medicines, Institute of Materia Medica, Chinese Academy of Medical Sciences and Peking Union Medical College, Beijing 100050, China. ^3^ Clinical Laboratory, The Affiliated Hospital of Qingdao University, Qingdao 266000, China. ^4^ Qingdao Hospital of Traditional Chinese Medicine, The affiliated Qingdao Hiser Hospital of Qingdao University, Qingdao 266033, China.

**Correspondence to:** Min Yang^1,2*^, State Key Laboratory of Digestive Health, Institute of Materia Medica, Chinese Academy of Medical Sciences and Peking Union Medical College, No. 2 Nanwei Road, Beijing 100050, China.

**E-mail:** minyang@imm.ac.cn

**Tel:** +86(10)63015871

# Supplemental Information

# Materials and methods

## Data sources and processing

Gene expression data and corresponding clinical information for colorectal cancer (CRC) were obtained from the Gene Expression Omnibus (GEO) database [1]. Multiple datasets were analyzed for different purposes. Differential expression of SEMA6A between CRC and adjacent normal tissues was evaluated using the GSE44076 dataset [2]. The prognostic values of SEMA6A and ISG15 were assessed in the GSE17538 cohort [3]. The correlation between SEMA6A and ISG15 expression was examined using three datasets: GSE29621, GSE38832, and GSE31595, with Spearman correlation coefficients calculated and scatter plots constructed [4-6]. Furthermore, the associations of SEMA6A and ISG15 with TGFB1 were investigated in seven independent datasets: GSE37182, GSE39582, GSE17538, GSE37892, GSE29621, GSE41258, and GSE38832 [7-10]. Using the "survMisc" R package’s cutp function, the optimal cutoff value for SEMA6A or ISG15 expression was determined, and patients with CRC were classified into high and low groups.

## Prognostic analysis and construction of the nomogram

To evaluate the predictive significance of SEMA6A in CRC, Kaplan-Meier (K–M) survival analysis was performed to compare survival outcomes across different patient groups. Univariable Cox regression analysis was then conducted to determine the hazard ratios for the SEMA6A groups and other clinical characteristics. Variables with a *P*-value < 0.05 were subsequently subjected to multivariable Cox regression analysis. Based on the results of the multivariable analysis, a Nomogram model was constructed to predict the 3- and 5-year overall survival (OS) of patients with CRC. The model's predictive accuracy was evaluated using calibration and receiver operating characteristic (ROC) curves. The "survival" and "regplot" R packages were used to generate the Nomogram, while "rms" and "timeROC" were employed to produce the calibration and ROC curves, respectively.

## Functional enrichment analysis

Differentially expressed genes (DEGs) across groups were identified using the "limma" R package, applying a filtering criterion of an adjusted *P*-value < 0.05 and an absolute log_2_(fold change [FC]) > 0.5. Functional enrichment analysis of the DEGs was performed, including Gene Ontology (GO) analysis and Gene Set Enrichment Analysis (GSEA), using the "clusterProfiler" R package [11].

## RNA sequencing (RNA-seq) and proteomics sequencing analysis

RNA concentration and purity were measured using a Nanodrop2000, RNA integrity was evaluated *via* agarose gel electrophoresis, and the RNA integrity number (RIN) was calculated using an Agilent2100. For library construction, the following RNA requirements were met: ≥ 1 μg of RNA, concentration ≥ 35 ng/μL, OD260/280 ratio ≥ 1.8, and OD260/230 ratio ≥ 1.0. To isolate mRNA, magnetic beads with oligo(dT) were used to capture poly-A-tailed mRNA through A-T base pairing, facilitating separation from total RNA. The mRNA was then fragmented using a fragmentation buffer into 300-bp-long pieces, after which a six-base random primer was added to reverse transcribe the mRNA into complementary DNA (cDNA). The cDNA was further processed by adding an "A" base to the 3' end to create a Y-shaped joint, and End Repair Mix was used to form blunt ends. Finally, sequencing was performed using the Illumina platform.

Proteomic quantification of the SL4 and pLV-SEMA6A cell lines was performed by Beijing YIKEBAIDE Technology Co., LTD. The cells were lysed at 4°C for 30 minutes, followed by centrifugation at 12,000g for 30 minutes at 4°C. The protein concentration of the supernatant was determined using the BCA assay. Tandem mass spectrometry (TMT) labeling was carried out according to the manufacturer's instructions (ThermoFisher, A44522). A 100 μg protein sample was dissolved in 100 mM TEAB, reduced with 100 mM TCEP at 37°C for 60 minutes, and alkylated with 40 mM iodoacetamide at room temperature for 40 minutes. To precipitate the protein, pre-cooled acetone was added to the reaction solution at -20°C and incubated for 4 hours. The protein was pelleted by centrifugation at 10,000g for 20 minutes, resuspended in 100 μL of 100 mM TEAB, and digested with trypsin overnight at 37°C. Acetonitrile was then added to the TMT reagent, and the digested protein was transferred to the TMT reagent and incubated at room temperature for 2 hours. The reaction was quenched with hydroxylamine for 30 minutes. Samples from the same group were combined, vacuum-dried, and subjected to one-dimensional separation *via* high-performance reversed-phase liquid chromatography (RPLC) prior to LC-MS analysis. The protein samples were reconstituted in loading buffer (2% acetonitrile, pH 10). A total of 10 fractions were collected and dried for subsequent MS analysis. The samples were recombined with a solution of 0.2% acetonitrile and 0.1% formic acid and analyzed by mass spectrometry.

Differential expression analysis was conducted using DESeq2. The R package "VennDiagram" was employed to visualize the overlapping differential genes between the transcriptome and proteome. Volcano plots of the differential genes were generated using the "ggplot2" R package. Target genes were screened and identified using cytoHubba, a plugin for Cytoscape software [12]. The top 10 genes from each of the 12 algorithms were selected, and the gene most frequently appearing across the algorithms was defined as the target gene of SEMA6A. GO functional enrichment analysis was performed using the "clusterProfiler" R package to identify potential regulatory pathways, and the co-enriched pathways from the transcriptome and proteome were visualized as bubble plots. Protein-protein and protein-pathway networks were constructed using ClueGO [13] and Cluepedia [14] in Cytoscape [15].

## Immune profile analysis and prediction of immunotherapy efficacy

Immune cell infiltration data for GSE17538 was obtained from the TIMER 3.0 database (Tumor IMmune Estimation Resource), which provides several deconvolution algorithms to assess the degree of immune cell infiltration, including Cell-type Identification By Estimating Relative Subsets Of RNA Transcripts (CIBERSORT) [16], Microenvironment Cell Populations-counter (MCP-counter) [17], Estimating the Proportion of Immune and Cancer cells (EPIC) [18], and TIMER [19]. The expression of various immune-stimulator genes across the groups was then compared. To predict the response to immunotherapy, the CheckMate 025 dataset [20] was subsequently used to evaluate the ability of SEMA6A combined with ISG15 to predict immunotherapy responses.

## Molecular docking analysis

The three‑dimensional structure of the human TGFβ1 protein (UniProt ID: P01137) was obtained from the UniProt database (entry AF‑P01137‑F1). The structure of human ISG15 (UniProt ID: P05161) was also retrieved from the UniProt database, corresponding to PDB entry 1Z2M. Both structure files were submitted to the ZDOCK server (version 3.0.2) for protein–protein rigid docking using standard mode and default search parameters, with the TGFβ1 set as the receptor and ISG15 as the ligand. After docking, the server generated the top 10 predicted complex conformations based on the ZDOCK score. The top‑ranked complex was selected and visualized using PyMOL.

## Statistical analysis

Statistical analyses were performed using one-way ANOVA or the Kruskal-Wallis test for comparisons between three or more groups, and the t-test or Wilcoxon test for comparisons between two groups. The chi-square test was used to assess differences in variable distributions among subgroups. Data visualization was carried out using R (version 4.1.3), and a *P*-value of less than 0.05 was considered statistically significant.

# Supplemental Figures


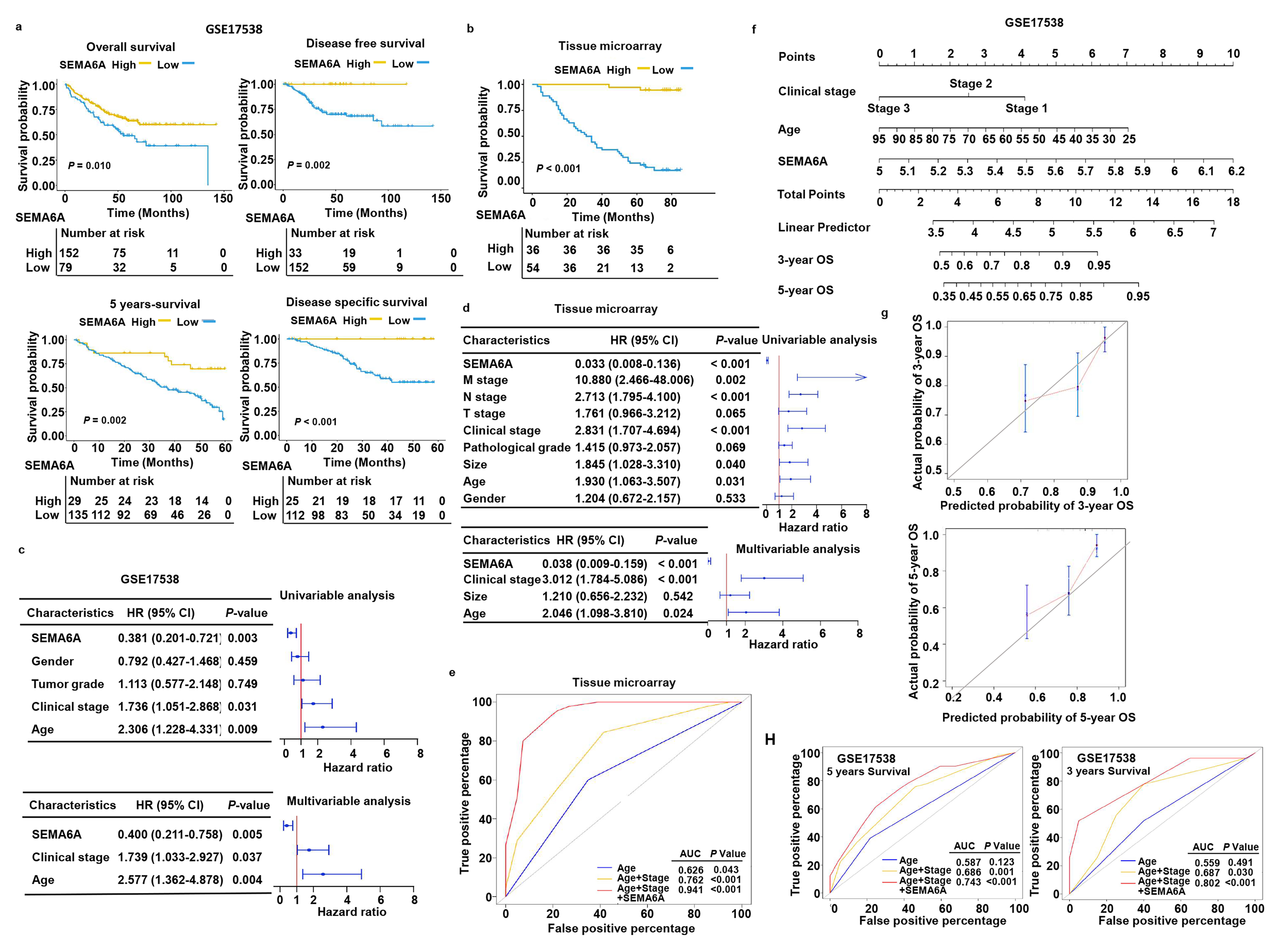


**Fig. S1:** Prognostic analysis and validation of SEMA6A in CRC. **a-b.** Kaplan-Meier survival analysis of overall survival, 5-year survival, disease-free survival, and disease-specific survival for SEMA6A in the GSE17538 dataset **(a)**, and tissue microarray **(b)**. **c-d.** COX regression analysis based on GSE17538 dataset **(c)**, and tissue microarray **(d)**. **e.** ROC curves for age, combined age and tumor stage, and combined age, tumor stage, and SEMA6A expression in TMA. **f-h.** Nomogram **(f)**, calibration curves **(g)**, and ROC curves **(h)** for the COX regression model based on the GSE17538 dataset.


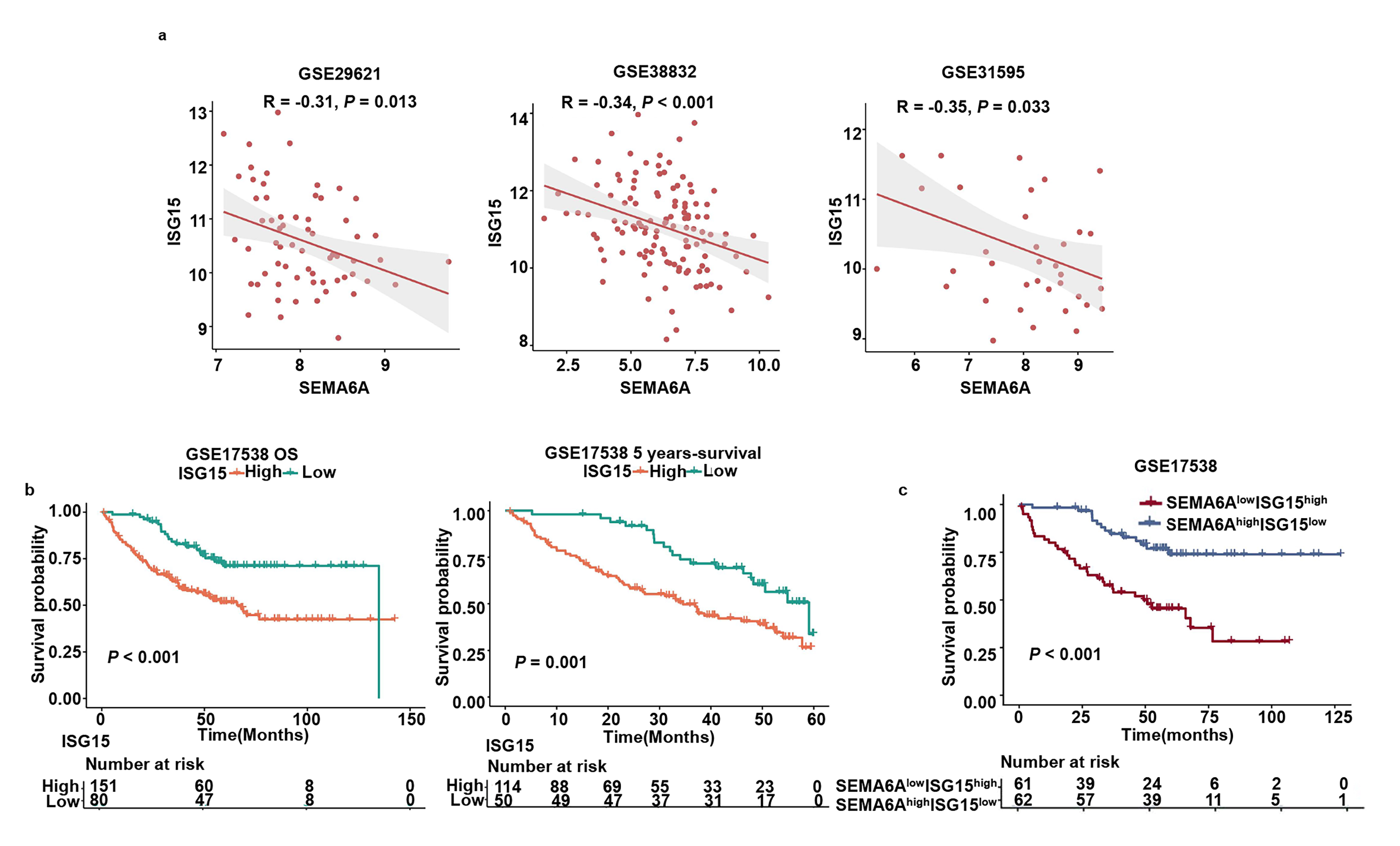


**Fig. S2:** Clinical correlation and prognostic significance of SEMA6A and ISG15 in colorectal cancer. **a.** Correlation between SEMA6A and ISG15 expression in the GSE29621, GSE38832, and GSE31595 datasets. **b.** Overall survival and 5-year survival curves for the ISG15 high- and low-expression groups in GSE17538. **c.** Overall survival curve for the SEMA6A^low^ISG15^high^ and SEMA6A^high^ISG15^low^ groups in GSE17538.


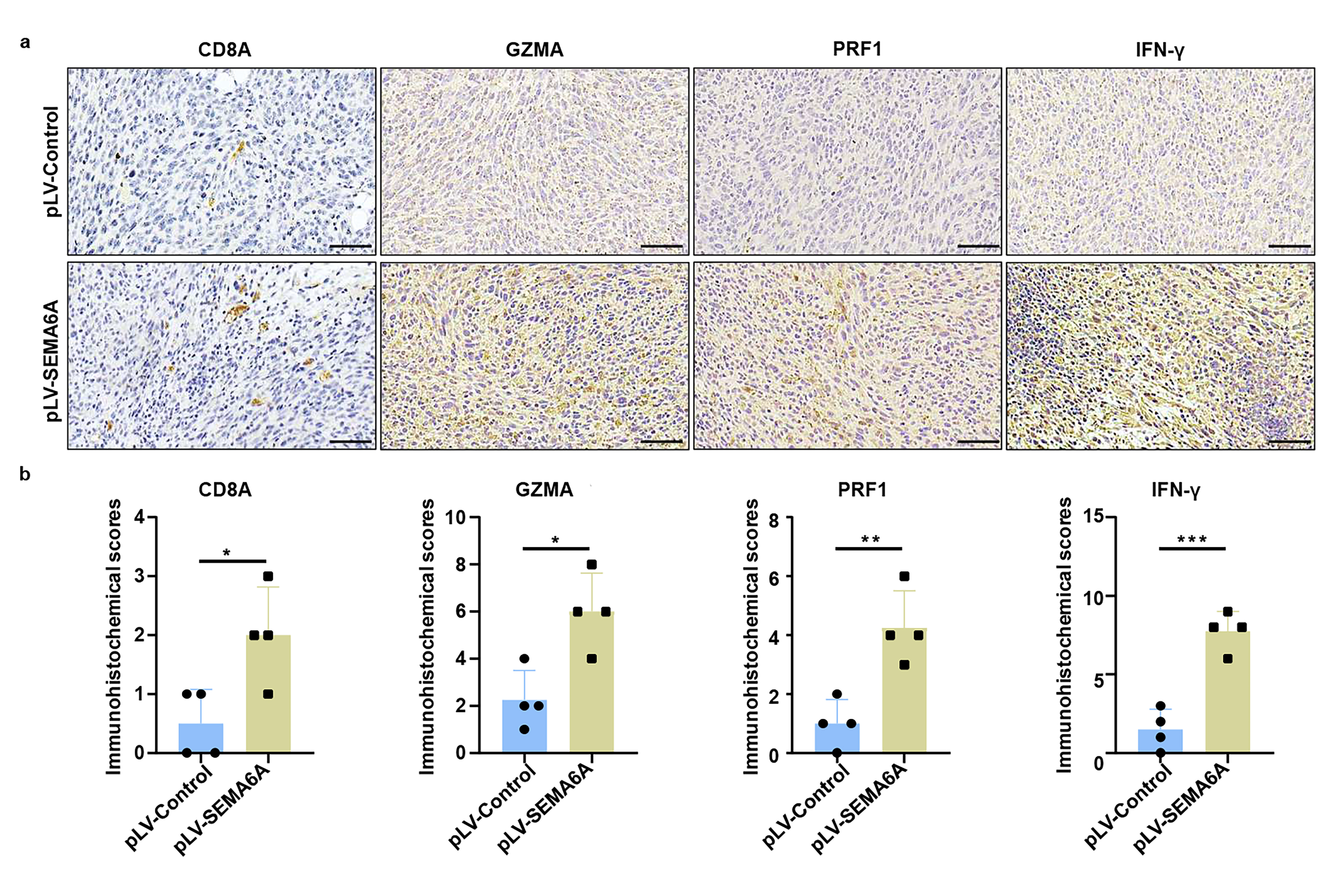


**Fig. S3:** SEMA6A overexpression promotes early CD8^+^ T cell infiltration and effector molecule expression in tumors. **a.** Representative IHC images of CD8A, GZMA, PRF1, and IFN-γ in tumor tissues on day 5 post‑inoculation (scale bar: 50 μm). **b.** Quantitative analysis of positive areas for the above markers. n=4. (**P* < 0.05, ***P* < 0.01, ****P* < 0.001)


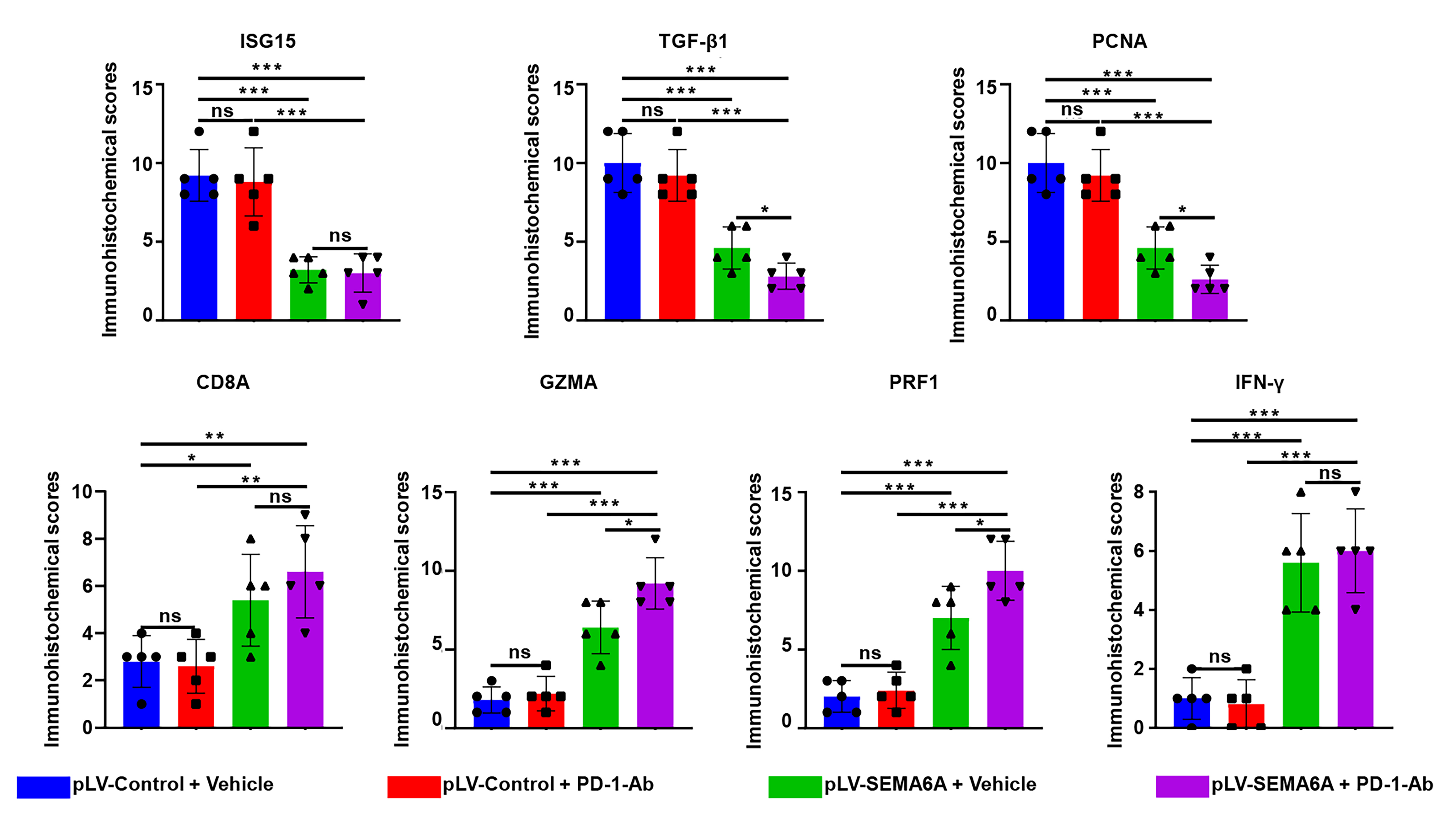


**Fig. S4:** Quantitative analysis of immunohistochemical staining in tumor tissues. (**P* < 0.05, ***P* < 0.01, ****P* < 0.001)

# Supplemental Tables

**Table S1** Antibodies used for WB, IF or IHC.

| Protein | Catalog Number | Company | Application |
| --- | --- | --- | --- |
| SEMA6A | ab154938 | Abcam | IHC (1:200),  WB (1:500) |
| β-actin | TA-09 | ZSGB-BIO | WB (1:1000) |
| Vimentin | BM0135 | Boster | IF (1:100) |
| E-cadherin | sc-8426 | Santa Cruz | IF (1:50) |
| ISG15 | 15981-1-AP | Proteintech | IHC (1:100),  WB (1:500) |
| GZMA-M | 11288-1-AP | Proteintech | IHC (1:100) |
| PRF1-M | 14580-1-AP | Proteintech | IHC (1:200) |
| IFN-γ-M | K009380P | Solarbio | IHC (1:100) |
| CD8A-M | ab217344 | Abcam | IHC (1:200) |
| PCNA-M | #2586 | Cell Signaling Technology | IHC (1:50) |
| TGFβ1-M | sc-130348 | Santa Cruz | IHC (1:50) |
| pSTAT3(Tyr705) | bsm-52211R | Bioss | WB (1:500) |
| STAT3 | bsm-52235R | Bioss | WB (1:500) |
| pSTAT1(Ser727) | bsm-52209R | Bioss | WB (1:500) |
| pSTAT1(Tyr701) | bsm-63180R | Bioss | WB (1:500) |
| STAT1 | bsm-63166R | Bioss | WB (1:500) |
| ISG15 | sc-166755 | Santa Cruz | WB (1:200), IP |
| FITC-CD8a | 100706 | Biolegend | FC (1:100) |
| PE-CD3e | 100301 | Biolegend | FC (1:100) |
| PE/Dazzle-CD45 | 103746 | Biolegend | FC (1:100) |
| APC-CD4 | 100515 | Biolegend | FC (1:100) |
| PE-CD69 | 104507 | Biolegend | FC (1:100) |
| APC-CD25 | 101909 | Biolegend | FC (1:100) |
| PE-TIM3 | 134003 | Biolegend | FC (1:100) |
| PE-CTLA4 | 106305 | Biolegend | FC (1:100) |

**Table S2** Primers used for RT-qPCR.

| Gene | Primer sequence |
| --- | --- |
| GAPDH F | CTGACTTCAACAGCGACACC |
| GAPDH R | TGAGCTTGA CAAAGTGGTCGT |
| SEMA6A F | CCCCACAACCCATCACTTCT |
| SEMA6A R | GGCACACTCGGGTTTATGGA |
| S100A F | AGAACAGTTGAGCAGACAGCC |
| S100A R | AGATGAGTTGCAGGCTTGGA |
| SNAIL1 F | GTCTGACCGATGTGTCTCCC |
| SNAIL1 R | TGTAAACATCTTCCTCCCAGGC |
| SNAIL2 F | GGCTCCCTCTTCCTCTCCATA |
| SNAIL2 R | GGGAATTCCATGGCAGTGAGA |
| TWIST F | AAAGGCATCACTATGGACTTTCTCT |
| TWIST R | ATGGTTTTGCAGGCCAGTTTG |
| ZEB1 F | CCCACTAGGAACAGGAACCAC |
| ZEB1 R | CTTATGCCAGGCACCCTGTT |
| ZEB2 F | TAGTCAGAGAAATGGCACCG |
| ZEB2 R | TCTCAAGACCAAATCGGAACA |
| FGF7 F | TCCTGGTTATCATGTGGTTGC |
| FGF7 R | GGAAGGATTCATCAAGAGAAGGGT |
| COL3A1 F | CTCCCATCAGAAAGATTCATTGGC |
| COL3A1 R | AAGCAGCCCCATAATTTGGTTT |
| VIMENTIN F | GCAGTTTTTCAGGAGCGCAA |
| VIMENTIN R | TCTTGTAGGAGTGTCGGTTGT |
| CTNNB F | TGGAACCTTGTTTTGGACAGTT |
| CTNNB R | AGCATCGTATCACAGCAGGTT |
| CXCL2 F | ACAGTGTGTGGTCAACATTTCT |
| CXCL2 R | CTCTGCTCTAACACAGAGGGAA |
| CD160 F | GCCCATAGCAGTATTTGGAGTC |
| CD160 R | ATCCTGCCCACTCTCTGCTT |
| IL6ST F | GGAAGCTCAGCCAACTCGAA |
| IL6ST R | CCCAAGCAGCCTTTCCATGA |
| LIF F | GTCTTGGCGGCAGTACACA |
| LIF R | GGAAGTCCGTCACGTTGGG |
| CSF1R F | CACCCCCATACTGGTACTGC |
| CSF1R R | CTAGCCCAGAATGACGGGAC |
| BTLA F | ACTGTTTGCCAAAATCACAAATCA |
| BTLA R | TGCATGTATGTCTCTGACACCA |
| ISG15 F | CGTGTTCATGAATCTGCGCC |
| ISG15 R | CTTGATCCTGCTCGGATGCT |
| GAPDH-M F | TGGCATTGTGGAAGGGCTCAT |
| GAPDH-M R | CAGCTTTCCAGAGGGGCCAT |
| LTA-M F | TCACCTCAGACAGGACCCAT |
| LTA-M R | AGCAGTGGCTGGCTTTTAGA |
| CD27-M F | AACACTACTGGACTGGGGGA |
| CD27-M R | AGAGAAGGAGGTGCCTGGTA |
| CD86-M F | AGCACGGACTTGAACAACCA |
| CD86-M R | CGTCTCCACGGAAACAGCAT |
| GZMA-M F | GGAGAGCCACGATGAGGAAC |
| GZMA-M R | AACAACCGTGTCTCCTCCAA |
| GZMB-M F | ACAACACTCTTGACGCTGGG |
| GZMB-M R | CGAGAGTGGGGCTTGACTTC |
| PRF1-M F | TCTTGGTGGGACTTCAGCTT |
| PRF1-M R | TGCTTGCATTCTGACCGAGT |
| GZMC-M F | ACACCTCCTTCCTCCCCTTC |
| GZMC-M R | GGAGAATCAGGACTGGTGGC |
| KLRD1-M F | ACAAGTGGGTTGGGCATCAG |
| KLRD1-M R | AGGCTGGAATTCTGCGAAGC |
| CD274-M F | CAGCAACTTCAGGGGGAGAG |
| CD274-M R | CGCACCACCGTAGCTGATTA |
| ISG15-M F | GATCAAGCATTTGCGCCTG |
| ISG15-M R | TTATAACCAACACTGGCTCTGG |
| ISG12-M F | GTCTTCCTGCACAGTGGACTT |
| ISG12-M R | CTAGGGGCATCTGAGTAGGGT |
| ISG20-M F | TGGGCCTCAAAGGGTGAGT |
| ISG20-M R | CGGGTCGGATGTACTTGTCATA |

# References

[1] Barrett T, Wilhite SE, Ledoux P, Evangelista C, Kim IF, Tomashevsky M, et al., NCBI GEO: archive for functional genomics data sets--update. *Nucleic Acids Res.* 2013;41(Database issue):D991-5. <https://doi.org/10.1093/nar/gks1193>.

[2] Sanz-Pamplona R, Berenguer A, Cordero D, Molleví DG, Crous-Bou M, Sole X, et al., Aberrant gene expression in mucosa adjacent to tumor reveals a molecular crosstalk in colon cancer. *Mol Cancer.* 2014;13(46. <https://doi.org/10.1186/1476-4598-13-46>.

[3] Smith JJ, Deane NG, Wu F, Merchant NB, Zhang B, Jiang A, et al., Experimentally derived metastasis gene expression profile predicts recurrence and death in patients with colon cancer. *Gastroenterology.* 2010;138(3):958-68. <https://doi.org/10.1053/j.gastro.2009.11.005>.

[4] Chen DT, Hernandez JM, Shibata D, Mccarthy SM, Humphries LA, Clark W, et al., Complementary strand microRNAs mediate acquisition of metastatic potential in colonic adenocarcinoma. *J Gastrointest Surg.* 2012;16(5):905-12; discussion 912-3. <https://doi.org/10.1007/s11605-011-1815-0>.

[5] Tripathi MK, Deane NG, Zhu J, An H, Mima S, Wang X, et al., Nuclear factor of activated T-cell activity is associated with metastatic capacity in colon cancer. *Cancer Res.* 2014;74(23):6947-57. <https://doi.org/10.1158/0008-5472.Can-14-1592>.

[6] Thorsteinsson M, Kirkeby LT, Hansen R, Lund LR, Sørensen LT, Gerds TA, et al., Gene expression profiles in stages II and III colon cancers: application of a 128-gene signature. *Int J Colorectal Dis.* 2012;27(12):1579-86. <https://doi.org/10.1007/s00384-012-1517-4>.

[7] Musella V, Verderio P, Reid JF, Pizzamiglio S, Gariboldi M, Callari M, et al., Effects of warm ischemic time on gene expression profiling in colorectal cancer tissues and normal mucosa. *PLoS One.* 2013;8(1):e53406. <https://doi.org/10.1371/journal.pone.0053406>.

[8] Marisa L, De Reyniès A, Duval A, Selves J, Gaub MP, Vescovo L, et al., Gene expression classification of colon cancer into molecular subtypes: characterization, validation, and prognostic value. *PLoS Med.* 2013;10(5):e1001453. <https://doi.org/10.1371/journal.pmed.1001453>.

[9] Laibe S, Lagarde A, Ferrari A, Monges G, Birnbaum D, and Olschwang S, A seven-gene signature aggregates a subgroup of stage II colon cancers with stage III. *Omics.* 2012;16(10):560-5. <https://doi.org/10.1089/omi.2012.0039>.

[10] Sheffer M, Bacolod MD, Zuk O, Giardina SF, Pincas H, Barany F, et al., Association of survival and disease progression with chromosomal instability: a genomic exploration of colorectal cancer. *Proc Natl Acad Sci U S A.* 2009;106(17):7131-6. <https://doi.org/10.1073/pnas.0902232106>.

[11] Yu G, Wang LG, Han Y, and He QY, clusterProfiler: an R package for comparing biological themes among gene clusters. *Omics : a journal of integrative biology.* 2012;16(5):284-7. <https://doi.org/10.1089/omi.2011.0118>.

[12] Chin CH, Chen SH, Wu HH, Ho CW, Ko MT, and Lin CY, cytoHubba: identifying hub objects and sub-networks from complex interactome. *BMC systems biology.* 2014;8 Suppl 4(Suppl 4):S11. <https://doi.org/10.1186/1752-0509-8-s4-s11>.

[13] Bindea G, Mlecnik B, Hackl H, Charoentong P, Tosolini M, Kirilovsky A, et al., ClueGO: a Cytoscape plug-in to decipher functionally grouped gene ontology and pathway annotation networks. *Bioinformatics (Oxford, England).* 2009;25(8):1091-3. <https://doi.org/10.1093/bioinformatics/btp101>.

[14] Bindea G, Galon J, and Mlecnik B, CluePedia Cytoscape plugin: pathway insights using integrated experimental and in silico data. *Bioinformatics (Oxford, England).* 2013;29(5):661-3. <https://doi.org/10.1093/bioinformatics/btt019>.

[15] Shannon P, Markiel A, Ozier O, Baliga NS, Wang JT, Ramage D, et al., Cytoscape: a software environment for integrated models of biomolecular interaction networks. *Genome research.* 2003;13(11):2498-504. <https://doi.org/10.1101/gr.1239303>.

[16] Newman AM, Liu CL, Green MR, Gentles AJ, Feng W, Xu Y, et al., Robust enumeration of cell subsets from tissue expression profiles. *Nature Methods.* 2015;12(5):453-457. <https://doi.org/10.1038/nmeth.3337>.

[17] Becht E, Giraldo NA, Lacroix L, Buttard B, Elarouci N, Petitprez F, et al., Estimating the population abundance of tissue-infiltrating immune and stromal cell populations using gene expression. *Genome biology.* 2016;17(1):218. <https://doi.org/10.1186/s13059-016-1070-5>.

[18] Xiao H, Zhang J, Wang K, Song K, Zheng H, Yang J, et al., A Cancer-Specific Qualitative Method for Estimating the Proportion of Tumor-Infiltrating Immune Cells. *Frontiers in immunology.* 2021;12(672031. <https://doi.org/10.3389/fimmu.2021.672031>.

[19] Li T, Fu J, Zeng Z, Cohen D, Li J, Chen Q, et al., TIMER2.0 for analysis of tumor-infiltrating immune cells. *Nucleic acids research.* 2020;48(W1):W509-w514. <https://doi.org/10.1093/nar/gkaa407>.

[20] Braun DA, Hou Y, Bakouny Z, Ficial M, Sant' Angelo M, Forman J, et al., Interplay of somatic alterations and immune infiltration modulates response to PD-1 blockade in advanced clear cell renal cell carcinoma. *Nat Med.* 2020;26(6):909-918. <https://doi.org/10.1038/s41591-020-0839-y>.
